# The nature of Pu-bearing particles from the Maralinga nuclear testing site, Australia

**DOI:** 10.1038/s41598-021-89757-5

**Published:** 2021-05-21

**Authors:** Megan Cook, Barbara Etschmann, Rahul Ram, Konstantin Ignatyev, Gediminas Gervinskas, Steven D. Conradson, Susan Cumberland, Vanessa N. L. Wong, Joёl Brugger

**Affiliations:** 1 School of Earth, Atmosphere and Environment, Monash University, Clayton, Australia; 2 Diamond Light Source, Harwell Science and Innovation Campus, Didcot, Oxon OX11 0QX United Kingdom; 3 Ramaciotti Centre for Cryo-Electron Microscopy, Monash University, Clayton, Australia; 4 Department of Chemistry, Washington State University, Pullman, WA USA; 5 Department of Complex Matter, Josef Stefan Institute, Ljubljana, Slovenia; 6 The University of Strathclyde, Glasgow, UK

**Keywords:** Materials science, Geochemistry, Mineralogy

## Abstract

The high-energy release of plutonium (Pu) and uranium (U) during the Maralinga nuclear trials (1955–1963) in Australia, designed to simulate high temperature, non-critical nuclear accidents, resulted in wide dispersion µm-sized, radioactive, Pu–U-bearing ‘hot’ particles that persist in soils. By combining non-destructive, multi-technique synchrotron-based micro-characterization with the first nano-scale imagining of the composition and textures of six Maralinga particles, we find that all particles display intricate physical and chemical make-ups consistent with formation via condensation and cooling of polymetallic melts (immiscible Fe–Al–Pu–U; and Pb ± Pu–U) within the detonation plumes. Plutonium and U are present predominantly in micro- to nano-particulate forms, and most hot particles contain low valence Pu–U–C compounds; these chemically reactive phases are protected by their inclusion in metallic alloys. Plutonium reworking was observed within an oxidised rim in a Pb-rich particle; however overall Pu remained immobile in the studied particles, while small-scale oxidation and mobility of U is widespread. It is notoriously difficult to predict the long-term environmental behaviour of hot particles. Nano-scale characterization of the hot particles suggests that long-term, slow release of Pu from the hot particles may take place via a range of chemical and physical processes, likely contributing to on-going Pu uptake by wildlife at Maralinga.

## Introduction

Plutonium (Pu)-bearing particles (0.45–2000 µm^1^) dispersed in the environment as a result of the nuclear fuel cycle, weapons testing and nuclear accidents are generally considered to be refractory and unreactive in their native state, but may be susceptible to chemical weathering and/or physical breakdown^2^. Plutonium can then become mobile and bio-available in water-soluble or particulate forms^3^, resulting in a long-term radiological risk to ecosystems and human health^1^.

At Maralinga, South Australia, the British nuclear testing program (1955–1963) involved seven nuclear weapons tests as well as numerous minor subcritical tests designed to investigate the performance of weapons components and safety issues^4^ (Table S1). The main contamination is due to the Vixen B trials that dispersed 22.2 kg of Pu and over 40 kg U across the 260 km^2^ ‘Taranaki’ test site^5^. Plutonium uptake by wildlife appears to have remained constant for the last ~ 30 years, leading to the suggestion of slow Pu release from hot particles as a result of on-going weathering at Maralinga^6^.

Maralinga hot particles were shown to be highly heterogenous based on bulk radionuclide contents and µm-scale chemical mapping of five particles by proton induced X-ray emission (PIXE) micro-spectrocopy^5,7^. This reflects the varied nature of the tests and associated devices^7^. Recently, Ikeda-Ohno et al.^2^ studied a single particle from the Vixen B trials using Synchrotron micro-X-ray fluorescence (µSXRF) and micro-X-ray absorption spectroscopy (µXAS); they confirmed that the Pu–U distribution was inhomogeneous, showed that Pu is present in the form of Pu(IV) (oxy)hydroxide compound(s), and speculated that the particle forms a "core–shell" structure with a Pu(IV) oxyhydroxide core surrounded by an external layer containing Ca, Fe, and U, which they linked to significant weathering of the particle following 50 years exposure.

This study aims to complement Ohno et al.’s^2^ study by (i) increasing the number of studied particles from one to six; (ii) avoiding the use of aqueous solvents in sample preparation to preserve delicate surface features and/or water-soluble forms of U/Pu; and (iii) extending the scope of characterization beyond chemical probes at µm-scale spatial resolution (µSRXF, µXAS) to µm-scale mineralogical (µXRD) and textural (µ-tomography), and to nano-scale textural and chemical characterization via dissection of selected particles with focused-ion beam-SEM (nominal resolution < 4 nm). This multi-scale characterization revealed considerable internal heterogeneity in the chemical and physical properties of all the investigated particles, resulting in a paradigm shift in our understanding of the nature of the Maralinga hot particles. We use the new information to (i) decipher the mechanisms that led to the formation of hot particles with highly complex and varied internal make-up at Maralinga; (ii) revisit the evidence for on-going weathering processes affecting Pu and U in the hot particles; a direct comparison of differential weathering of Pu and U is made possible by the fact that both actinides coexist in these particles; and (iii) explore the pathways for long-term (decades to 100’s of years) Pu release from the hot particles. The Maralinga particles are models for hot particles released during subcritical nuclear incidents, and are also useful in nuclear forensics as proxies for those generated from dirty bombs^8^. Overall, these new results corroborate that particle inhomogeneity and morphology^1,2,9^ are as or even more important as the speciation as determinants of the fate of U and Pu in the environment, and demonstrate that the accuracy of predictive models depends on the inclusion of these parameters obtained from experimental measurements.

## Non-destructive bulk characterization of hot particles

**Figure 1:**
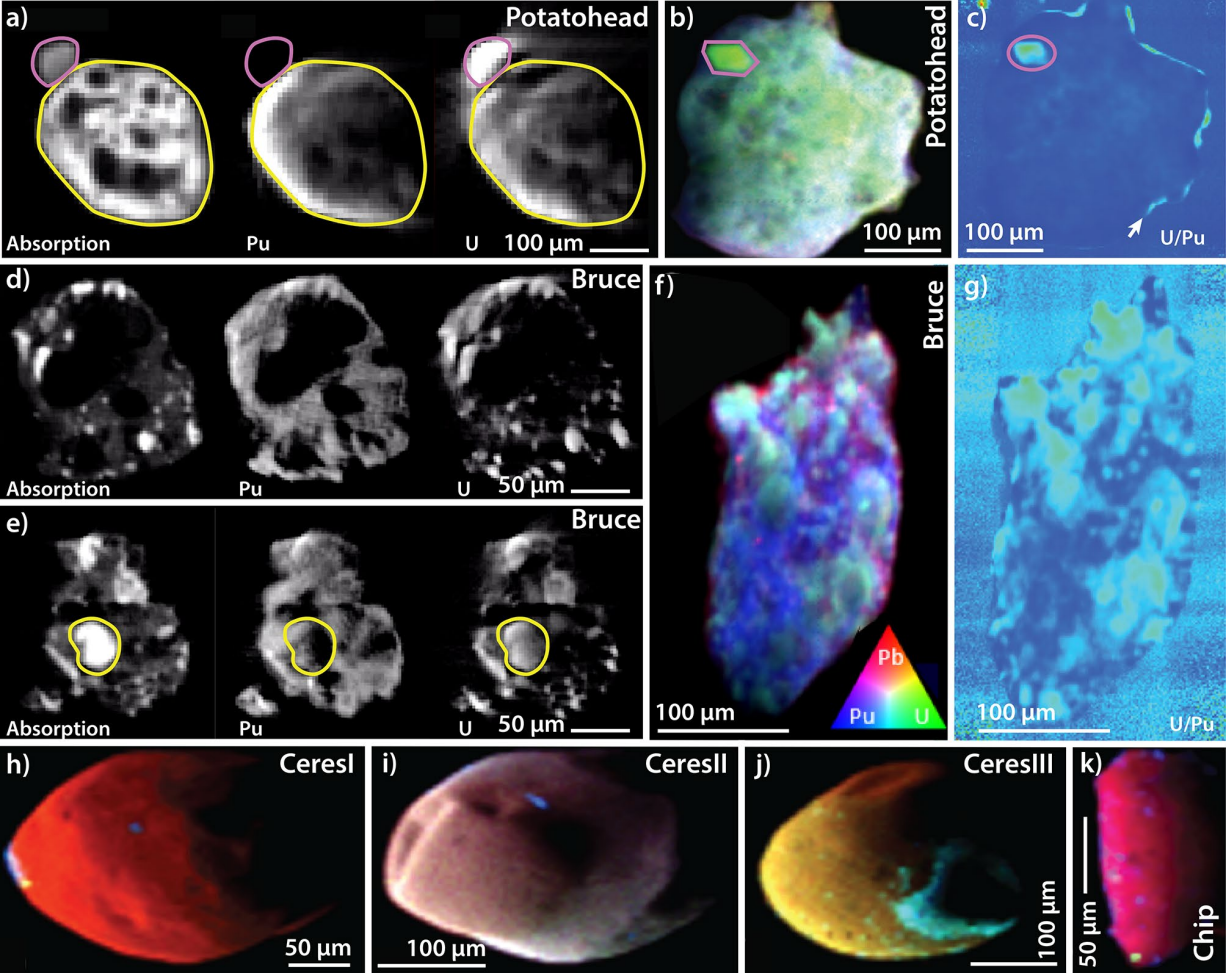
X-ray µ-tomography (**a**,**d**,**e**) and µSXRF imaging (**b**–**c**, **f**–**i**) of hot particles from Maralinga. In (**a**) (*Potatohead*) the low-X-ray cross-section areas within the particles (black) correspond to pores, whereas in (**d**) and (**e**) (*Bruce*) they correspond to Al-oxide rich domains. *Potatohead* displays a U-rich grain (purple circle in **a**,**b**,**c**) attached on the outside of the particle; in (**e**), a U-rich, Pu-poor inclusion is highlighted in yellow.

The six particles (Fig. 1) were extracted from proximal and distal soils collected at Taranaki ground zero (Fig. S1) in 1984 during remediation efforts, and archived since then at the Australian Radiation and Nuclear Safety Agency in sealed containers under ambient conditions (details in Supplementary Information (SI-Materials and methods). The particles named *Potatohead* and *Bruce* were extracted by Burns et al.^5,7^, whereas *CeresI*, *CeresII*, *CeresIII* and *Chip* were extracted during this study from archived soil samples. A dry extraction procedure ensured minimal perturbation of the surface of the particles during their recovery from the soils (details in SI-Materials and methods). The particles are classified into three different chemical groups based on µSXRF data (Table 1; Fig. 1; elemental maps in Fig. S2; bulk spectra in Fig. S3): (i) relatively homogeneous co-location of Pu + U (*Potatohead* from the NW plume; Fig. 1b,c), (ii) highly heterogenous distribution of Pu + U (*Bruce* from the NE plume; Fig. 1f,g) and (iii) Pb-rich particles with discrete Pu ± U inclusions (*CeresI–III* + *Chip* from the northern plume; Fig. 1h–k).

**Table 1:** Summary of hot particle characterization results using µ-SXRF, µ-XAS, and µ-XRD techniques.

Particle name Locality	Description	Major elemental composition	Oxidation state of Pu, U present	Major mineral composition
*Bruce* (GNE*) North-east plume	Irregular, uneven oblong particle with bumpy surface. Length = 518 µm	Pu, U, Fe, Al	Pu(0) + Pu(IV) ± Pu(III), U(IV/VI)	Pu–U-C, PuO_2±x_, UO_2±x_/U_3_O_7,_ Al–Fe alloy/Al_2_O_3_
*Potatohead* (ZD600*) North-west plume	Irregular trapezoidal shape with uneven surface. Length = 336 µm	Pu, U, Al, Fe	Pu(IV), U(IV/VI)	PuO_2±x_, UO_2±x_/U_3_O_7_
*CeresI* (K480i) 100 m north GZ	Spherical, slightly oval particle with smooth surface containing slight imperfections. Length = 218 µm	Pb, Pu, U	Pu(IV), U(IV)	Native lead
*CeresII* (K480iI) 100 m north GZ	Irregular, somewhat spherical particle with square protrusion on surface. Length = 216 µm	Pb, Pu, U	n/a	Native lead
*CeresIII* (K480iii) 100 m north GZ	Spherical particle with smooth surface containing slight imperfections. Length = 222 µm	Pb, Pu, U	n/a	Native lead
*Chip* (K480iv) 100 m north GZ	Irregular, oblong particle with smooth surface. Length = 110 µm	Pb, Pu, U	n/a	Native lead

*Particles from study of Burns and co-workers^5,7^.

**Figure 2:**
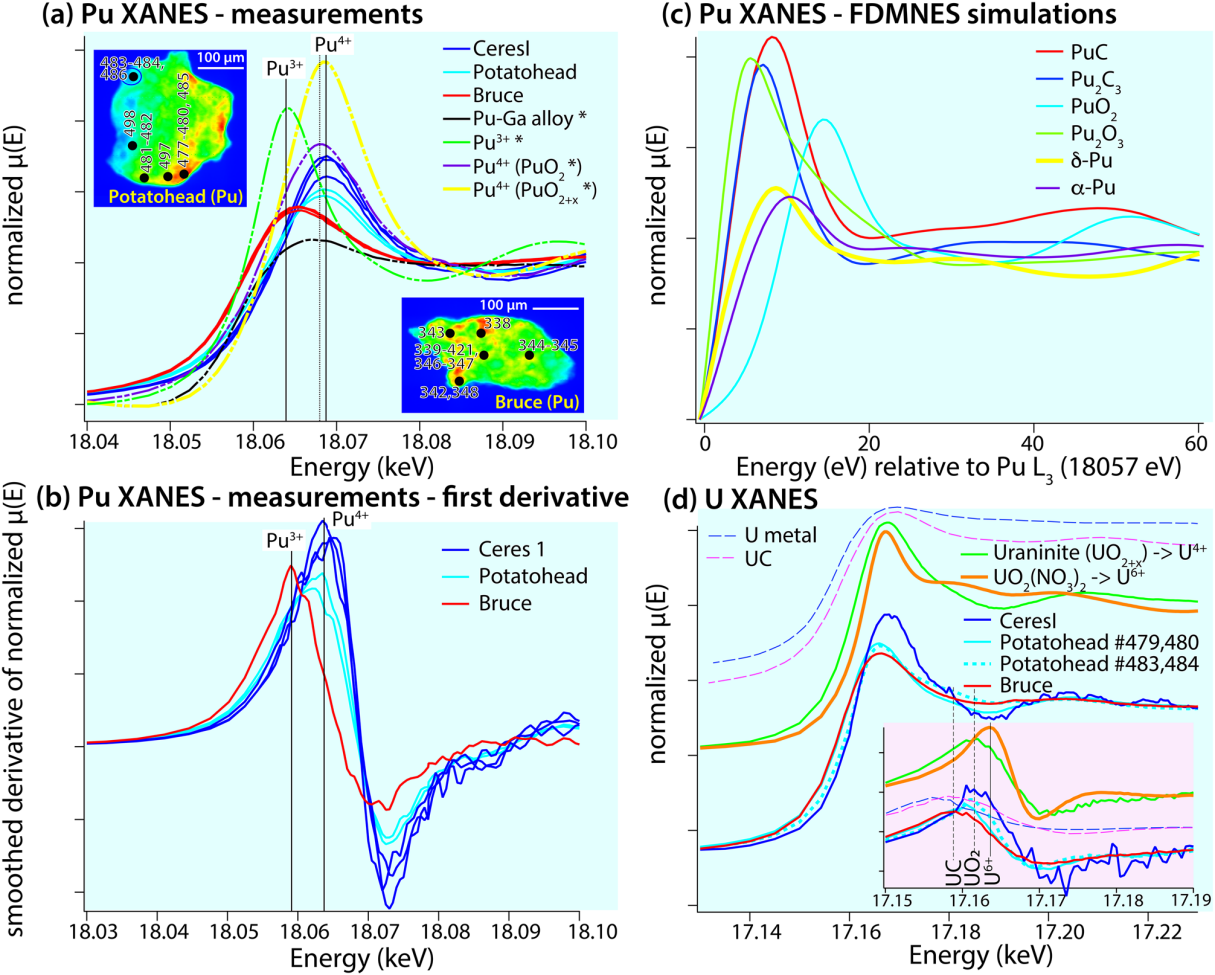
Pu and U µXANES of the *Potatohead*, *Bruce* and *CeresI* hot particles. (**a**) Pu µXANES of particles, compared to Pu XANES standards (*) from Conradson et al.^10,50^; locations of the spectra are shown in the Pu L_3_ µSXRF maps, together with analysis numbers; (**b**) derivatives of the data shown in (**a**); (**c**) *first principle* FMDNES simulations of Pu spectra. PuO_2_ was calculated to check that the simulations can reproduce known spectra and then these simulations were used to calculate Pu-carbide spectra, demonstrating that these have a white line at lower energy than PuO_2_. Crystal structure data: PuC^51^; Pu_2_C_3_^52^; PuO_2_^53^; Pu_2_O_3_^54^; δ-Pu^55^; α-Pu^56^. (**d**) U µXANES of particles compared to uraninite (predominantly U^4+^) and uranyl nitrate (U^6+^). Inset shows the first derivative of the µXANES spectrum. The spectra of metallic U and UC as taken from^13^.

*Potatohead* and *Bruce* were further characterised by µSXRF-fluorescence tomography (Fig. 1a,d,e), µ-X-ray Absorption Near Edge Structure (µXANES) (Fig. 2), µ-Extended X-ray Absorption Fine Structure (µEXAFS) (Fig. S4) and µ-X-Ray Diffraction (µXRD) (Fig. S5, S6) to provide information on Pu–U oxidation states and identify mineral phases present in the particles (details in SI-Hot particle composition).

### Potatohead: single Pu form

This particle was found to closely resemble the particle studied by Ikeda-Ohno^2^: XANES spectra show that Pu is present mainly as Pu(IV) (Fig. 2) and the bond lengths derived from EXAFS shell-by-shell fits (Table S2; Fig. S4a) are typical of those for PuO_2±x_. Similarly, µXANES and µEXAFS results indicate that U is present mainly as U(IV), consistent with the (U,Pu)O_2±x_ phases. The µXRD data, however, indicate that *Potatohead* is made of two isomorphic crystalline phases with distinct compositions, (Pu,U)O_2±x_ and (U,Pu)O_2±x_ (Fig. S5; Table S3). The U XAS results also show that a U-rich, idiomorphic grain on the outside of the particle, circled in purple in Fig. 1a–c, contains a high proportion of U(VI) in the form of uranyl (short U=O bond distances (1.76–1.78 Å) from EXAFS data, Table S4, analyses #483–484 and #486; XANES spectra showing a significant edge shift to higher energy relative to the bulk grain, and a uranyl-characteristic band at ~ 17.18–17.19 keV, Fig. 2d).

### Bruce: various forms of Pu

**Figure 3:**
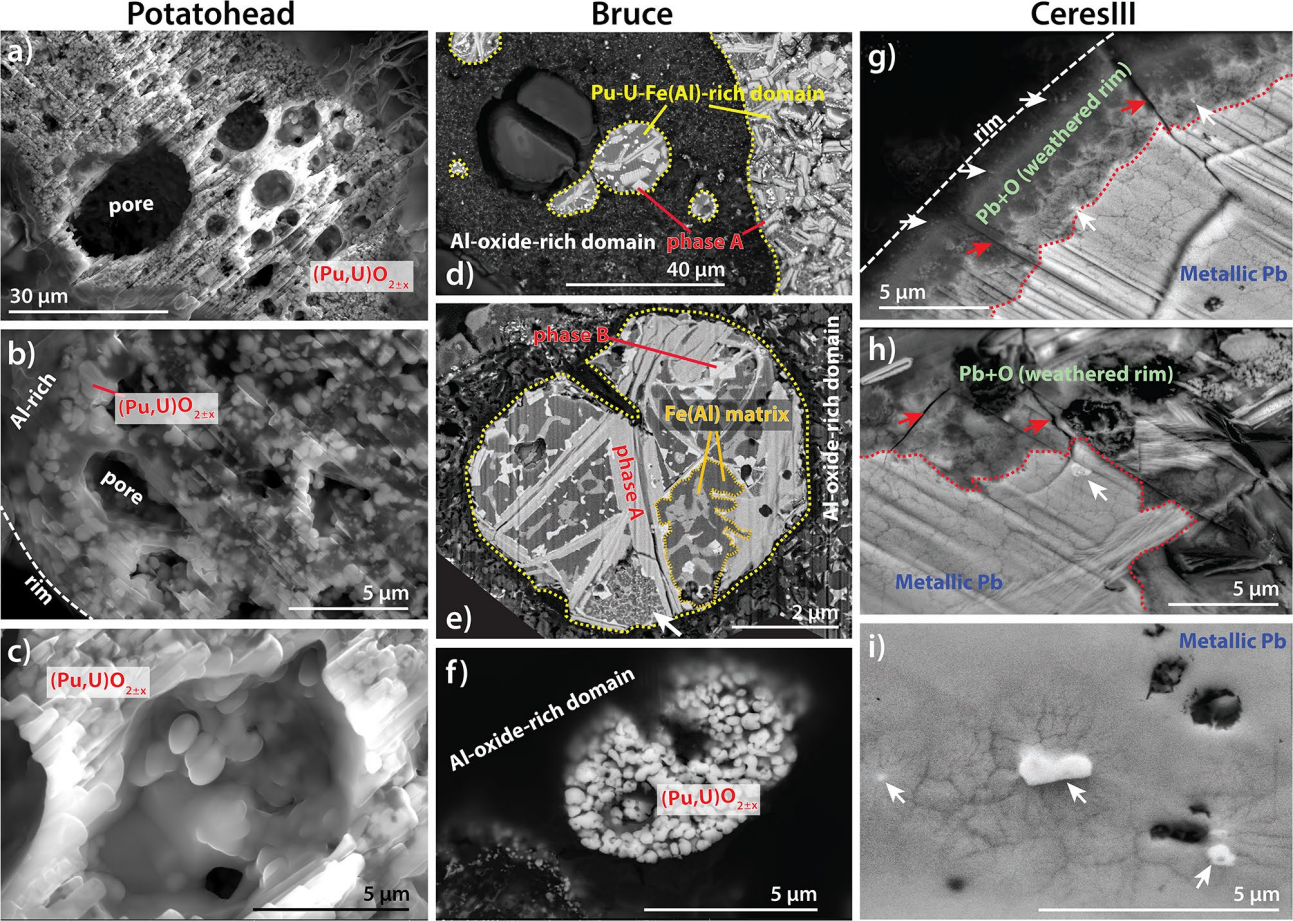
Imaging (BSE mode) of fresh FIB cut surfaces within the hot particles *Potatohead*, *Bruce* and *CeresIII*. Domains and phases are labelled based on EDS spot analyses and maps. (**a**) Popcorn-like texture of (Pu,U)O_2±x_ phase(s) and pores due to gas entrapment. (**b**) Nano-sized (Pu,U)O_2±x_ particles in an Al-oxide-rich matrix on the edge of the particle. (**c**) Detail showing absence of weathering product within a pore. (**d**) Textures suggestive of coexistence of Al-oxide-rich and Pu–U-Fe(Al)-rich melts. (**e**) Detail of Pu–U-Fe(Al)-rich aggregate. (**f**) Small aggregate of (Pu,U)O_2±x_ nano-crystals. (**g**) Rim of the particle, showing oxidation of the metallic lead. (**h**) Detail of the weathering rim. Arrows in (**g**,**h**) indicate Pu-rich micro- to nano-particles. (**i**) Fracturing around Pu-carbide inclusions (white) resulting from radiation damage.

**Figure 4:**
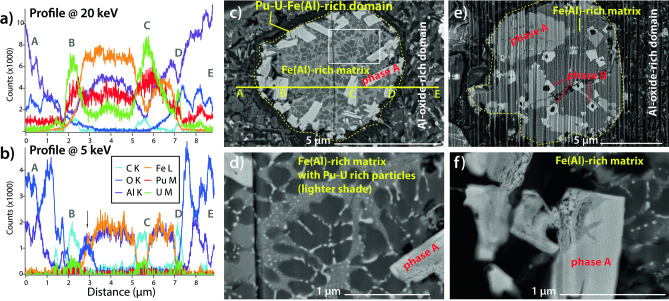
Details of the composition of *Bruce*. (**a**,**b**) FIB-SEM EDX line scans across the Pu–U-Fe(Al)-rich spheroid shown in (**c**); both profiles were measured at the same location, but at 20 and 5 kV respectively. These line scans demonstrate that the outer phase (labelled Al-oxide-rich matrix) consists predominantly of Al-O, and contains low levels of Pu–U, both in the background (site labelled ‘A’) and in particulate form (‘E’). The large, light grey, lath-shaped phase A grains (sites ‘B’, ‘C’, ‘D’) are Pu–U–C with minor Fe–Al. The matrix within the spherical area contains variable amounts of Fe–Al: the lighter areas contain more Fe than Al, i.e. between B and C, compared to the darker area that contains more Al than Fe, i.e. between C and D. This Fe(Al) alloy matrix also contains low levels of Pu–U. (**d**) Detail of (**c**); location in (**c**) is marked by a white rectangle. The Fe(Al) matrix of the this Pu–U–Fe(Al)-rich spheroid has broken down into an assemblage of two Fe(Al) alloys with different Fe:Al ratios (dark- and medium grey), and Pu–U rich nanoparticles (white). (**e**) Another Pu–U–Fe(Al)-rich sphere that contains phase A and B inclusions; note vapor bubbles associated with phase B. Vertical curtailing is an artifact of FIB polishing. (**f**) A detail of phase A, showing local compositional variation and porosity.

µXRD data reveal a complex and heterogeneous phase make-up, including (Pu,U)O_2±x_ + (U,Pu)O_2±x_, bromellite (BeO), and Al–Fe and Al–Mg alloys (Fig. S6). Together, the Pu-XANES and electron microscopy results provide strong evidence for the presence of low valence Pu compounds in *Bruce*. The Pu µXANES spectra (Fig. 2a,b) are shifted to a lower energy compared to Pu(IV), indicating contribution of a lower valence state, but the white line is less intense and broader than that expected for Pu(III) compounds^10,11^. These spectra can be fitted as a linear combination of Pu(0) + Pu(IV). FIB-SEM results (Figs. 3, 4) indicate that Pu occurs in at least three different forms within *Bruce*, with the first two likely associated with low valence Pu rather than Pu(III): (i) carbide phases, (ii) a minor constituent in Fe-Al alloys, and (iii) Pu–U oxides. The presence of Pu carbides has not been reported in environmental particles to date. Dyker and Bertrand^12^ note that carbon exists in metal-like state in some carbides, an observation that is confirmed by the XANES spectrum of uranium carbide (UC) resembling a combination of metallic U and UO_2_ spectra^13^; indeed, the U XANES spectrum obtained from *Bruce* indicates a lower formal oxidation state of U compared to UO_2_, and is consistent with a significant proportion of U present in carbide and/or in metallic-like (alloy) form (Fig. 2d). In the absence of published XANES data on Pu-carbides, we calculated the XANES spectra of known compounds using the ab-initio code FDMNES^14,15^; these simulations confirm that the white lines of Pu-C compounds are at lower energy than in PuO_2±x_ (Fig. 2c).

### *CeresI–III* + Chip

**Figure 5:**
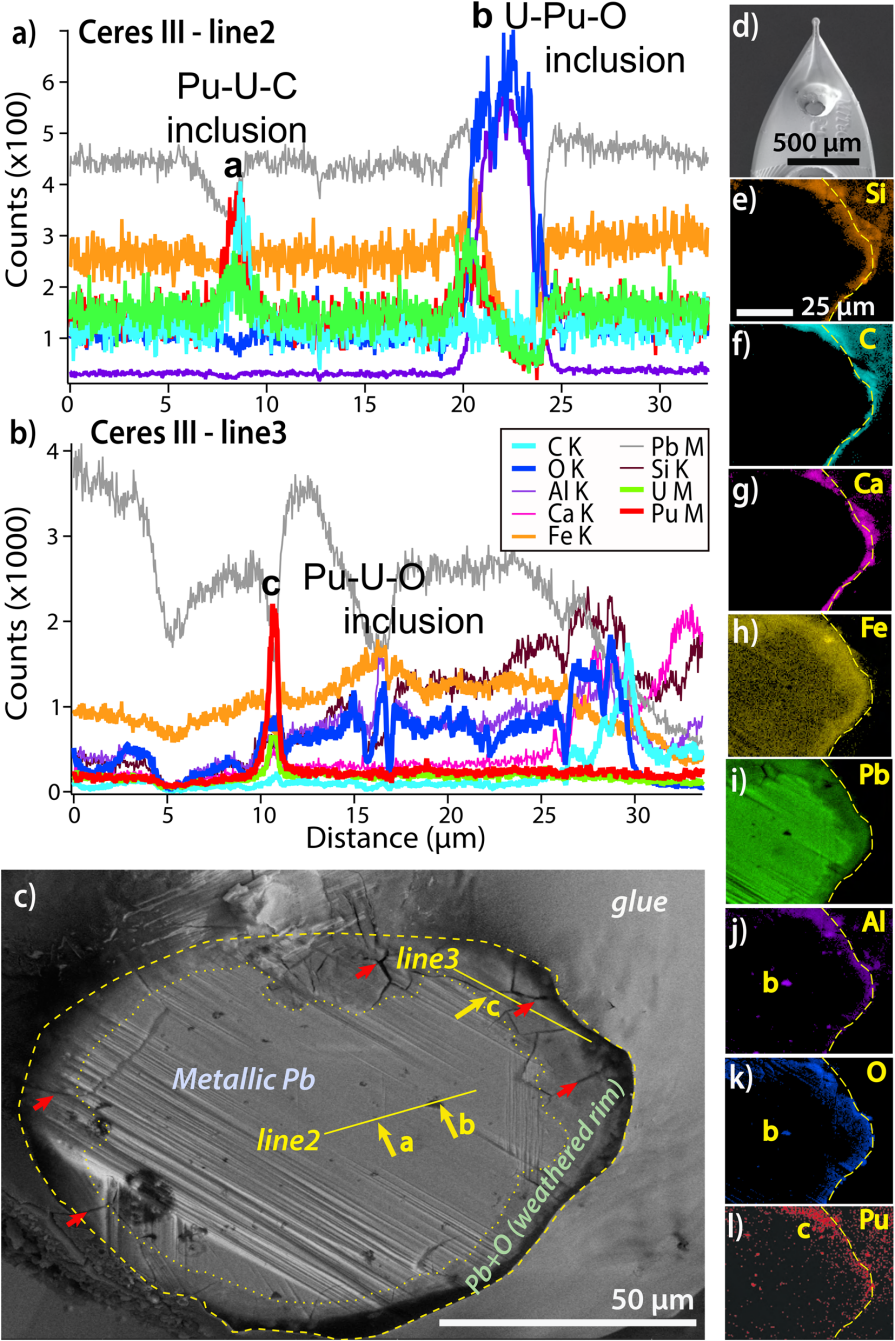
FIB-SEM EDX characterization of *CeresIII*. (**a**,**b**) FIB-SEM EDX line scans of selected areas within *CeresIII*. (**c**) SEM image of FIB cross-section of the particle. (**d**) Overview of the particle mounted on kapton micromount. (**e**–**l**) Represent false colour EDS element maps for each respective element. This particle is predominantly metallic Pb, with some Fe and traces of Pu–U in the center; there are also a couple of Pu–U–C particles (at sites labelled ‘a’ and ‘b’, line 2). There is a rim of Pb–O, which contains a particle comprised of Pu–U–O (arrow and site ‘c’, line 3) and an outer rim containing Ca–C–O (most likely calcite, CaCO_3_) and Si (most likely a silica polymorph). Imaging and EDS analyses performed at 20 keV acceleration voltage.

These four particles consist predominantly of metallic Pb (Figs. 3g-i, 5). Plutonium and U are present in particulate form (arrows in Figs. 3g–i, 5), with different Pu:U ratios (different colours in Fig. 1h–k; this is confirmed by the correlation plots shown in Fig. S2g). XANES data indicate that Pu exists mainly as Pu(IV), and the U XANES spectra closely match those of uraninite (i.e., UO_2+x_, Fig. 2d).

µSXRF-fluorescence tomography data further highlight the differences between *Potatohead* and *Bruce* (Fig. 1a,d,e; full movies in SI). The concentrations of U and Pu are generally positively correlated in *Potatohead* (Fig. S2g), with the exception of the U-rich grain on the outside (Fig. 1a–c), but they are decoupled in *Bruce*: the framework is Pu-rich, but *Bruce* also contains particles of a U-rich phase varying in size from ≤ 2 to 10 µm (Fig. 1d,f,g; Fig. S2g). Both particles contain volumes characterised by low total X-ray attenuation (Fig. 1a,d,e); FIB-SEM data (see below) reveal that these volumes correspond to empty pores in *Potatohead*, but consist of light elements (Al-oxide-rich domains) in *Bruce*.

## Dissection of the hot particles reveals extreme textural complexity

The characteristic X-rays of light elements are fully absorbed by less than a µm of U/Pu-rich material, which limits the information that can be gathered via non-destructive X-ray fluorescence. Hence, FIB-SEM was used to dissect *Potatohead*, *Bruce*, *CeresI* and *CeresIII* (Figs. 3, 4, 5; a gallery of images is included in SI Fig. S7). High resolution SEM imaging and X-ray spectroscopy of the freshly exposed surfaces reveal the significant complexity of the internal textures of the grains and the micro (< 5 µm) to nano-particulate (1–450 nm) nature of the Pu-rich phases; we follow the definition of nano-particle of Salbu et al.^1^.

### Potatohead

The (Pu/U)O_2±x_ phases in *Potatohead* exist as particles that are up to ~ 1 µm in diameter, and are embedded in an Al-rich matrix (Fig. 3a–c). The FIB-SEM imaging also shows that the porosity observed via X-ray tomography consists of abundant spherical pores with sizes down to ~ 1 µm. The morphology of the pores is indicative of gas entrapment; (Pu,U)O_2±x_ is highly volatile compared to (U,Pu)O_2±x_, with boiling at 1 bar observed at temperatures as low as 2300 K and compositions as rich as 50 mol% UO_2_^16^. All compositions in the UO_2±x_–PuO_2±x_ binary are melted above ~ 3120 K, and melts can persist down to ~ 2700 K^16^. The presence of two distinct (Pu,U)O_2±x_ phases revealed by XRD (Fig. S5b) most likely relates to crystallization of *Potatohead* from immiscible U- and Pu-rich melts, as a complete solid solution exists below 2000 K^16^.

### Bruce

FIB-SEM revealed a level of chemical and textural heterogeneity not previously recognised in Maralinga hot particles (Figs. 3d–f, 4). The textures indicate the coexistence of at least two immiscible polymetallic melts during the formation of the particle in the explosion environment: spherical inclusions of Pu–U–Fe(Al)-rich composition float in an Al-oxide-rich matrix (Fig. 3d, SEM–EDX maps in Fig. S8). The Al-oxide-rich domains correspond to the low-density areas in the X-ray tomograph (Fig. 1d,e), and also contain numerous, sub-µm-sized inclusions of a Pu–U-rich phase (light spots in the Al-oxide rich domains in Fig. 3d,e). Aggregates (5–10 µm in diameter) of nano-particulate (Pu/U)O_2±x_ similar in texture and composition to *Potatohead* also occur within the Al-oxide-rich matrix (Fig. 3f). Texturally, the Pu–U–Fe(Al)-rich domains consist of Pu–U-rich, lath-shaped crystals up to ~ 10 µm in length (Phase A: medium grey in BSE on Figs. 3d,e and 4c–f), often decorated with ~ 1 µm-sized, cube-shaped crystals (Phase B; Figs. 3d, 4e) and embedded in an Fe(Al)-rich matrix (shades of dark grey in BSE, Figs. 3d,e, 4e,f). Based on the combination of XANES spectroscopy, SEM-FIB EDS line profiles (Fig. 4a–c; additional example shown in Fig. S9) and SEM-FIB EDS point spectra (Figs. S10), we interpret Phase A and B to be carbides, with Phase A also possessing Al as an essential constituent (Fig. 4a,b). Phase A commonly contains cracks and pores (Figs. 3e, 4f). Small (≤ 600 nm) spherical pores, most likely gas bubbles, are also associated with some Phase B crystals (Fig. 4e). The matrix of these Fe(Al)-rich domains may be inhomogeneous with varying Fe:Al ratios in an eutectic textural relationship typical for cooling of small liquid droplets^17^ (Figs. 3e, 4c,e). These Fe-Al alloys also show evidence for sub-solidus decomposition, resulting in fine grained textures with two different Fe-Al alloys and numerous Pu–U nanoparticles (< 100 nm in diameter) (arrow in Fig. 3e; detail in Fig. 4d).

The FIB-SEM cuts (Fig. 5d) confirm that *CeresIII* (Fig. 5) and *CeresI* (Fig. S11) consist of metallic Pb, with minor Fe and Cu contents. Texturally, *CeresI* consists of lamellae ≤ 5 µm in width and > 30 µm in length, with the matrix containing more Fe and Cu than the lamellae (Fig. S11). This internal texture is consistent with formation via cooling of melts. In this case, the melts are dominated by Pb, which was a major constituent of the featherbeds used at Taranaki^18^. *CeresI* shows no evidence of weathering, but *CeresIII* displays a thin (5–10 µm thick) rim of Pb–O replacing metallic Pb (Figs. 3g,h, 5c,k), indicating localised oxidation (weathering). Inclusions (< 2 µm) of Pu–U-carbide are scattered throughout *CeresIII* (Figs. 3i, 5a); within the Pb–O weathering rim, the Pu-rich particles are finer (< 1 µm) and consist of Pu–U-oxides (Figs. 3g,h, 5b). Two types of porosity are present: the Pb–O weathering rim contains numerous cracks that extend into the metallic lead core (red arrows in Figs. 3g,h, 5c); and cracks surrounding Pu–U-carbide inclusions within unaltered metallic Pb are suggestive of radiation damage (Fig. 3i).

## Discussion

### Evolving mobility pathways

**Figure 6:**
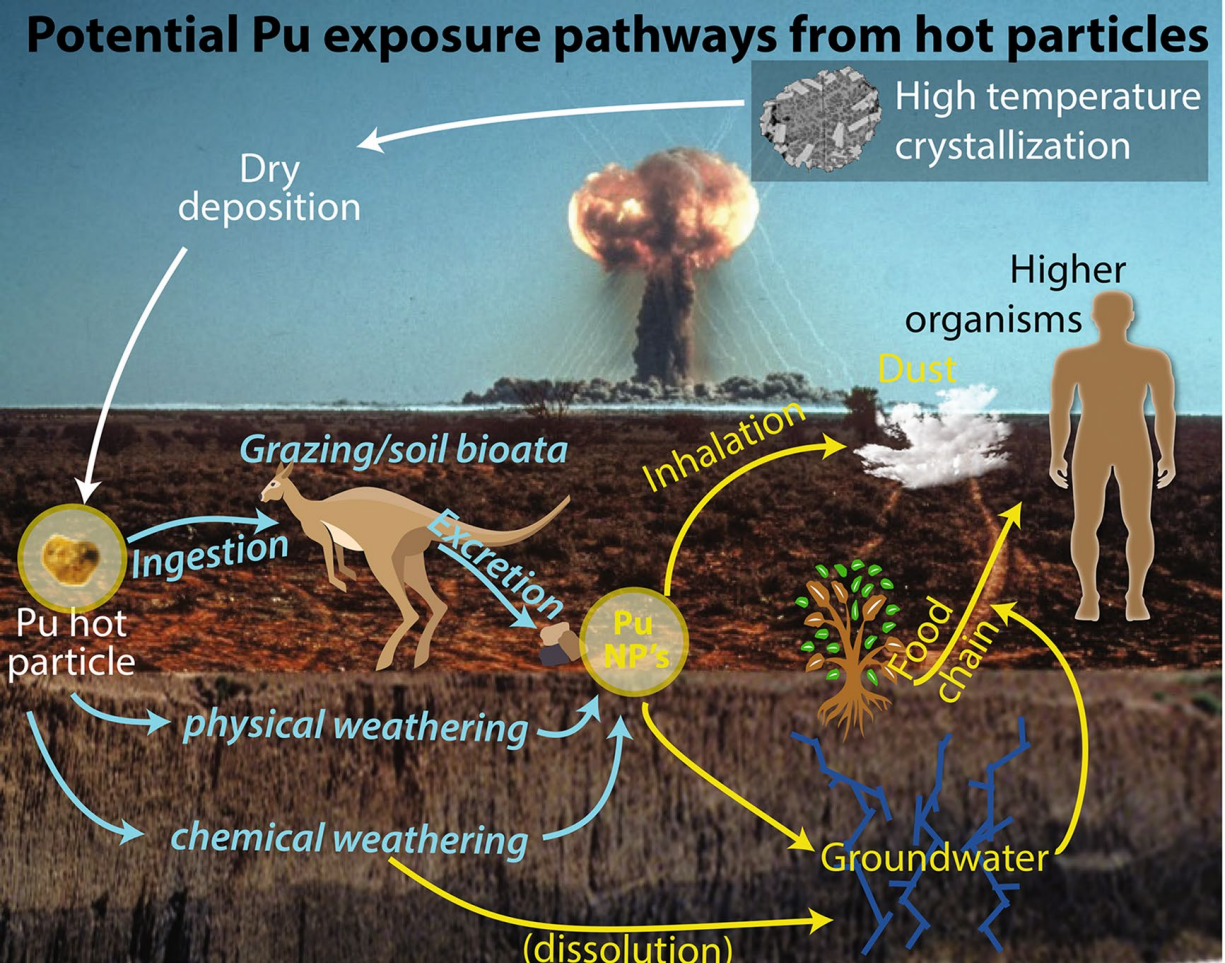
Inferred exposure pathways at Maralinga: the nature of hot particles influences Pu cycling in the environment. The colours in the figure reflect: processes in explosion cloud (white), breakdown processes of Pu particle (blue), exposure pathways via mobile phases (yellow). The background image is from the UK Atomic Energy Authority/Atomic Weapons Research Establishment, available under the Open Government License (http://www.nationalarchives.gov.uk/doc/open-government-licence/version/3/) and National Archives of Australia A6456, R075/004..

Liberation of Pu from hot particles was proposed to play a significant role in explaining the continuous exposure of animals at Maralinga^6^. Previous micro-characterization of Maralinga hot particles was limited to non-destructive µm-scale studies via PIXE and µXRF/µXAS^2,5,7^. *Potatohead* is similar in texture (fragile, highly porous) and composition (Pu present mainly in (Pu,U)O_2±x_ like form) to the particle studied by Ikeda-Ohno^2^; however, our multi-scale and multi-method characterization of diverse hot particles invites a revisit of the implications of earlier results for the fate of Pu at Maralinga: our results reveal a great variability in the nature and internal make-up of hot particles; they provide direct evidence for decoupling of U and Pu geochemistry during weathering at Maralinga; and identify that some particles contain a significant amount of Pu in low-valence state, which is unexpected for particles that survived for ~ 30 years in the environment. Here, we start by discussing the significance of these results with respect to the chemical and physical factors, summarised in Fig. 6, that contribute to Pu liberation from the hot particles. Such observations provide a mechanistic foundation for predicting the future evolution of the particles and likely exposure pathways^6^. Finally, we emphasize how the nature of the particles is a direct result of the source material and their mode of formation^1^, which is revealed to be via cooling of polymetallic melts by textural and compositional data, and we discuss the broader implications of these results for high temperature sub-critical nuclear incidents.

### Physical breakdown

The different particles have contrasting bulk composition and internal make-up; these differences probably reflect differences in the source term of individual tests^7^ and correlates with the locations of the particles (Fig. S1). Maralinga hot particles have long been recognised to be friable; for example, Ikeda-Ohno’s^2^ particle broke into smaller pieces during gentle handing, as did our *Chip* particle. The FIB-SEM data reveal that this fragility can result from a combination of factors. (i) *Bruce* and *Potatohead* are intrinsically friable as a result of their chemical and physical heterogeneity down to the nm-scale. This phase heterogeneity results in joints and fractures providing points of mechanical weakness within the particle. Potatohead also features a high amount of porosity (Figs. 1a, 3a). (ii) Further mechanical weakening and fracturing are caused by chemical weathering, which induces fracturing in the Pb-rich particles (Figs. 3g,h, 5c). (iii) Finally, radiation damage also contributes to fracturing and increasing porosity (Fig. 3i). ^239^Pu (weapons grade Pu used during Vixen B consisted of ~ 95% ^239^Pu; Table S1) generates 3 × 10^9^ α-particles g^−1^ s^−1^; the porous halos around the Pu-rich particles extend radially around 5 µm (Fig. 3i), suggesting that each 5 meV alpha particle travels ~ 5 µm in the Pb-rich matrix causing damage along its path^19^. Phase A also shows internal fracturing (Fig. 3e) and porosity (Fig. 4f), that could be due to relaxation of internal stress during cooling, chemical weathering, or radiation damage.

Mechanical weathering is expected to dominate in deserts; the Maralinga nuclear test site is an arid environment (monthly mean temperatures range from 13 to 25 °C), with cool winters with overnight frosts (minimum − 3 °C), hot summers with temperatures frequently exceeding 40 °C, and large diurnal fluctuations in temperature^6,18,20^. In this context, the fact that Pu is mostly hosted in phases with particle size that range from ~ 5 to < 100 nm means that physical weathering will release particles that can travel in dust (particles less than 7–10 µm are identified as a respirable risk) or in groundwater. In addition to increasing mobility, the reduction in size of Pu particles increases the radiological risks significantly, as the probability of exposure increases, since α-emitters such as Pu need to be located at the surface of the particle to cause an immediate radiological risk; hence, the effective dose increases as the size of the particles decreases. The increasing surface area with decreasing particle size also enhances the susceptibility to chemical weathering.

### Chemical weathering

Our results confirm the mostly refractory nature of Pu in the hot particles^1^. This is in contrast with U, which shows significant in-particle mobility. Some of the U in *Potatohead* is in the form of uranyl (U(VI)O_2_^2+^) (Fig. 2d), and µSXRF maps show evidence for some U redistribution in *Potatohead* in the form of (i) a rim enriched in U relative to Pu (arrow in Fig. 1c) and a U(VI)-rich crystal growing on the outside of the particle (Figs. 1a–c, 2d). In general, U(IV) phases are insoluble at room temperature, and U mobility is mainly related to U(VI) uranyl complexes (e.g., aqua, carbonate, sulfate, and chloride^21^). Similarly, Ikeda-Ohno et al.^2^ suggested that U was leached out of the single particle they studied, based on µSXRF data showing Ca + U enrichment on its periphery. Based on µSXRF and µXANES (predominance of (Pu,U)O_2±x_) data, *Potatohead* is similar to Ikeda-Ohno et al.’s^2^ particle; however, the FIB-SEM data show that *Potatohead* does not show the core–shell structure inferred by Ikeda-Ohno et al.^2^. Since the high-resolution SEM images do not show evidence of hydrous weathering, even along grain boundaries or inside pores (Fig. 3b,c), U oxidation and mobilization likely must result from limited capillary action through this porous particle. Calcium most probably reflects precipitation of calcite (‘calcrete’)^22^ on the outside of the particle, rather than Ca substitution of U/Pu during (U,Pu)O_2±x_ leaching. Similarly, EDS maps of the *CeresIII* particle show an enrichment in Ca–C–Si on the outside of the particle, reflecting a thin coating of calcite (Ca,C) and some silicate/clay mineral (Si) (Fig. 5e–l).

A core–shell style chemical weathering was observed in *CeresIII* (Figs. 3g–i, 5), replacing the Pb alloy with a Pb–O phase. This oxidation increased porosity, and Pu speciation changed from low-valence Pu in the core to Pu–O compounds in the weathered rim, concomitant with a decrease in size of the Pu-rich particles. However, despite these changes in Pu speciation, it appears that little or no Pu was released from the particle (e.g., Fig. 5l).

Further, a significant portion of Pu is present in low valence form in *Bruce* and *CeresIII*. Although uranium carbide (UC) has been found in depleted U particles from Kosovo and Kuwait^23^, this is the first report of Pu-carbide and Pu-bearing alloys in hot particles retrieved from the environment. Low valence Pu–U-carbide phases are typically pyrophoric at µm-grain size in contact with water or molecular oxygen^24^, yet these phases were retained despite ~ 30 years exposure to an arid environment and then another ~ 30 years stored at ambient conditions. These phases were probably protected by their inclusion in Fe–Al alloy (*Bruce*) and metallic Pb (*CeresIII*), as well as by the presence of impurities. In *CeresIII*, Pu–U-carbide exists within metallic Pb, but a Pu–U-oxide is predominant within the oxidised rim (Fig. 5c). The properties of actinide carbides are strongly dependent on minor or trace elements in their crystal structure^24^; this may further stabilize the carbide phases. Indeed, there was no evidence of oxidation of the Pu–U-carbide phases in Bruce following exposure to atmospheric, ambient conditions for around 10 months (Fig. S12). Hence, Pu-rich phases can persist in chemical states that are far from equilibrium with their environment (water, air), as a result of the physical and chemical make-up of the hot particles.

### Ingestion and modification by stomach acids

Aside from mechanical breakdown and chemical weathering by exposure to moisture and atmosphere, hot particles may be transformed via ingestion by animals^6,20^. Leaching tests using 0.16 M HCl to simulate stomach acids found that just ~ 0.20% of ^239+240^Pu was leached in ~ 200 h from a millimeter-sized particle with a glassy structure from Ground zero, Semipalatinsk, former USSR^1^, in line with the expected refractory nature of Pu hot particles. However, similar tests on six particles recovered from Taranaki (containing < 20 wt% PuO_2_; other metals present in significant concentrations were U, Fe, Cr) found solubilities varying from 1 to 96% over 40 days^5^. Note that compared to the simple dissolutions tests conducted by^1,5^, stomach acids also contain enzymes that will affect hot particle behaviour, but the FIB-SEM results can explain the wide range in behaviour in HCl-leaching experiments: (i) although (Pu,U)O_2±x_ would be poorly soluble in these tests, dissolution of the Al-oxide-rich domains binding the individual µm- to nm-sized (Pu,U)O_2±x_ grains in *Potatohead*-type particles may release the Pu-rich grains, some of which have sizes < 0.45 µm; similarly dissolution of Al- and Fe-rich phases in *Bruce*-type particles would release Pu-rich nanoparticles. Dissolution of the Pb/PbO-rich particles is limited by the low solubility of PbCl_2_(s).

### Particulate transport

As summarised in Fig. 6, the weathering processes discussed above may result in the release of Pu-rich particles ≤ 5 µm from the hot particles. As Pu exists in micro- to nano-particulate form in all studied particles, despite their different chemistries, Pu liberation does not require a chemical process; simple mechanical breakdown will release nanoparticles. All released Pu-rich particles cause an inhalation risk, and the finer fraction (nanoparticles) can be transported into the groundwater during rainfall events. Nano-particle-facilitated transport of Pu in groundwater was first identified at the Nevada test site^3,25^ and more recently at (i) the weapon-producing sites of the Mayak Production Association, Urals, Russia^26^; Rocky Flats, Colorado, USA^27^ and Hanford, Washington, USA^28^; (ii) at the nuclear power sites at Sellafield/Windscale, UK; Chernobyl, Ukraine; and Marcoule, France^28^; and (iii) in rivers waters related to the Grimsel test site, Switzerland^28^. The arid environment at Maralinga is conductive to both dust and water-borne nano-particulate transport; although rainfall is limited (~ 240 mm/year), it is often of a convective nature and characterised by high-intensity, short-duration events (≥ 3 days with > 10 mm rainfall per year^29^). Although direct evidence of nano-particle transport is still lacking at the ultra-dilute conditions at Maralinga, direct imaging of the nature of the Pu-rich phases in the hot particles and their behaviour during weathering suggest that this may be a primary cause for wild-life exposure^6^.

### From fire to dust

Once liberated from the hot particles, the environmental behaviour of Pu is governed by complex processes^3,30^ involving solubility, hydrolysis, complexation and sorption (with both inorganic and organic ligand and phases^31,32^) and nano-particle (colloid) formation reactions (all possibly catalysed by microbiota^33^). Yet, the potential for Pu to migrate through the soil environment and enter the food chain, and the resulting risk to biota (Fig. 6), can be estimated using radioecological models. These models need to consider not only the amounts of radioactive material released to the environment following an accident, but also the physical and chemical characteristics of the contaminant and their potential changes through time in order to determine the long-term impacts arising from contamination^1,3,34–37^. Based on non-destructive micro-analytical characterization, hot particles from sub-critical nuclear incidents across the globe are chemically and texturally heterogeneous (Table S5). This heterogeneity is hindering their inclusion in radioecological models that are used to predict long-term risks^38^.

At Maralinga, the particles contain Pu (and U) in the form of high temperature, anhydrous phases, that are far from equilibrium with respect to environmental conditions. Textural and phase relationship considerations reveal that all studied particles formed via cooling of polymetallic melts resulting from fissile material mixing with the hot detonation environment. The Pb, Fe and Al present in the particles reflect the composition of the individual devices and detonation characteristics. Unfortunately, no information is available in public records on the specifics of the designs and materials of individual tests. Most Pu is hosted in nano-phases that crystallised during the cooling of these polymetallic melts, and, consequently, the micro- to nano-particulate nature of the Pu in these hot particles, regardless of their bulk composition, is an intrinsic result of their formation via cooling of micro-droplets of polymetallic melt^17^ (Figs. 3d, 4). The hot, anhydrous micro-environment under which the particles condense in the explosion cloud also accounts for the crystallization of phases that contain Pu (and U) in low valence state (carbides; Pu in Fe-(Al)-alloys). Sub-solidus reactions (e.g. *Bruce*; arrow in Figs. 3e, 4c,d) and weathering (*CeresIII*, Figs. 3g,h, 5) further contributed to the generation of fine Pu-rich nanoparticles (< 100 nm) subsequent to cooling.

The recognition of the nature and internal make-up of the hot particles has important consequences for the cycling of Pu in the arid environment at Maralinga. As summarised in Fig. 6, liberation of micro- to nano-particulate Pu is promoted over time via mechanical breakdown, facilitated by the heterogenous and/or highly porous nature of the particles and thermal cycling in the environment. In addition, near-surface particles have a high probability of ingestion by soil biota and higher animals. Dissolution via digestion of the reactive, metallic matrix of the particle (Fe-, Al-, or Pb-rich alloys) could result in the further release of Pu-rich nanoparticles. Compared to the parent hot particles, these micro- (≪ 5 µm) and nanoparticles have additional toxicity risks due to their small sizes, the low-valence state of Pu and their mobility in dust and groundwater.

The Vixen B trials at Maralinga were designed to simulate sub-critical nuclear incidents, i.e. accidents where energy is released from external fires or conventional explosives. Since these tests, the world has documented a few instances of such incidents (Table S5, SI-Previous studies), including the B-52 accidents that resulted in the conventional detonation of thermonuclear weapons near Palomares, Spain^39^ and Thule, Greenland^39,40^; and the explosion of an armed nuclear missile and subsequent fire at the McGuire Air Force Base, USA^9^. Hot particles from these sub-critical nuclear incidents share the chemical and textural heterogeneity of the Maralinga particles as a result of the similar mode of formation from polymetallic melts. In particular, the distinctive texture of *Potatohead* (Fig. 3a–c) is remarkably similar to that of the particles from Palomares^39^ and Thule^40^; and hot particles from the McGuire Air Force Base accident have µSXRF images and Pu–U correlations similar to either *Bruce* and *Potatohead*^9^. In summary, hot particles released via high energy sub-critical incidents acquire their compositions and textures at high temperature within the explosion cloud, and this mode of formation sets the scene for their long-term environmental behaviour^1^. We note that hot particles released following high energy failure of containment in nuclear reactors, such as Chernobyl^17,41,42^ and Fukushima^43^, also show heterogeneous compositions, with U ± Pu associated with Zr and other metals from the reactor cladding^43–45^. These particles share a formation via cooling of high temperature melts, however in this case melting occurs in the nuclear fuel rather than in the explosion cloud. In addition, aerodynamic silica fallout particles generated in near surface critical nuclear tests were found to contain a heterogeneous distribution of elements, related to environmental and device material being incorporated and fractionated in the fireball^46–48^.

Between 1950 and 1988 alone, there were more than 230 recorded nuclear weapon accidents, including at least 10 with documented release of radioactive particles into the environment^49^. The risks of such incidents are only increasing as international treaties such as the *Intermediate-Range Nuclear Forces Treaty* and *NewSTART* come to an end. Yet, there is currently no international best practice for the inclusion of Pu–U rich hot particles released during sub-critical incidents in environmental impact assessment or risk characterization. The new observations on the hot particles from the Maralinga tests provide a clear explanation for the heterogeneous behaviour of different hot particles with respect to chemical and physical weathering^5,41^ that has hindered predictive modelling. Bulk characterization of the hot particles provides limited information about the nature and heterogeneity of the particles^2^. This issue is alleviated by the use of FIB-SEM; nano-scale chemical and textural characterization exposes the diversity of hot particles; allows identification of the different weathering pathways in historic particles; and provides the fundamental information for predicting the future behaviour of the particles and the radioecological risk to humans and non-human biota.

## Supplementary Information

Supplementary Information.

Supplementary Video 1.

Supplementary Video 2.

Supplementary Video 3.

## Data Availability

All data is available in the main text or the supplementary materials.
