# Supplementary material for: The nature of Pu-bearing particles from the Maralinga nuclear testing site, Australia
Source: Sci Rep. 2021 May 21;11:10698. doi: 10.1038/s41598-021-89757-5 (PMC8139974; doi:10.1038/s41598-021-89757-5)
Supplement: Supplementary file 1 — Supplementary Information. [file 41598_2021_89757_MOESM1_ESM.pdf]

Revision 1

Supplementary Materials for

The Nature of Pu-bearing particles from the Maralinga Nuclear Testing Site Australia

Authors: Megan Cook[1], Barbara Etschmann[1], Rahul Ram[1], Konstantin Ignatyev[2], Gediminas Gervinskas[3], Steven D. Conradson[4], Susan Cumberland[5], Vanessa N.L. Wong[1], Joël Brugger[1]

*Correspondence to: barbara.etschmann@monash.edu; joel.brugger@monash.edu

Materials and Methods. 3

Sample Site Description. 3

Soil sampling and dry extraction of hot particles. 3

Data collection at Diamond Light Source. 5

SXRF data processing. 6

XRD data processing. 6

XANES and EXAFS data processing. 7

Simulation of XANES spectra. 7

Fluorescence tomography data processing. 8

FIB-SEM. 8

Hot particle composition. 9

Potatohead: single Pu form. 9

Bruce: various forms of Pu. 9

FIB-SEM – line scans of Bruce. 10

CeresI: predominantly Pb with heterogenous distribution of Pu + U. 12

Previous studies. 13

Supplementary Figures. 14

Figure S1. Geographical location of Maralinga. 14

Figure S2. µSXRF characterisation of hot particles from Maralinga. 15
Figure S3. µSXRF spectra of the six hot particles. 16
Figure S4. Results of µEXAFS analysis at Pu and U L_3edges. 17
Figure S5. µXRD results for *Potatohead* (data shown as solid lines, fits as dashed lines). 18
Figure S6. µXRD patterns of *Bruce*. 19
Figure S7. FIB-SEM images of *Bruce* (1 to 21), *Potatohead* (22-27) and *CeresIII* (28-42). 20
Figure S8. FIB-EDX elemental maps of *Potatohead* and *Bruce*, collected at 24 kV. 23
Figure S9. FIB-SEM EDX line scans across a Pu-U-Fe rich 'blob' within *Bruce*. Data were collected at 20 kV acceleration voltage. 24
Figure S10. FIB-SEM point spectra analysis within *Bruce*. 25
Figure S11. Composition and texture of the *CeresI* hot particle. 26
Figure S12. FIB-SEM images of *Bruce* taken ~10 months apart, showing relative stability of the contained phases upon exposure to air. 27

Supplementary Material: Tables. 28

Table S1: A history of British nuclear tests in mainland Australia. The studied particles originate from the Vixen B series of trials. 28
Table S2. Particle Pu-L_3 edge EXAFS refinements. 32
Table S3. Qualitative µXRD TOPAS refinement of *Potatohead*. 33
Table S4. Particle U-L_3 edge EXAFS refinements. 34
Table S5. Previous studies characterising Pu-bearing particles/colloids with imaging and/or XAS techniques. The studies are grouped by their broad geographical location. 35

Supplementary Movies. 39

Movie S1: *Potatohead*. The reconstructed volume, in orange, corresponds to the pores and the blue corresponds to the envelope of the grain. 39
Movie S2: *Bruce* (a) slice from Fig. 1c and (b) slice from Fig. 1d. The reconstructed volume, in orange, corresponds to the Al-oxide-rich low-density phase and the blue corresponds to the envelope of the grain. 39

References. 39

Materials and Methods.

Sample Site Description.

The first contamination of the Maralinga (South Australia) test site with plutonium hot particles resulting from nuclear detonations occurred in September 1956 as part of the British nuclear weapons' program[1]. Seven nuclear weapons tests and five series of minor trials totalling 588 tests (Table S1) investigating the performance of various components of a nuclear device and their sensitivity to accidents (e.g., plane crash) took place as part of the British Nuclear Testing Program between 1952 and 1963 (Table S1). The secrecy surrounding the program at the time of the detonations and poor record keeping resulted in an incomplete account of the tests performed during the minor trials [1]. The incomplete account of these minor trials creates difficulty in linking particle characteristics to specific sources and in turn the prediction of interactions in the environment and a full understanding of environmental impacts and risks. Burns et al. [2] note that the $^{239}Pu/^{241}Am$ and $^{240}Pu/^{241}Am$ signatures of Potatohead and Bruce, in conjunction with meteorological conditions, are consistent with those expected from the Vixen B trials.

The trial site at the focus of this study, Taranaki, is 30 km north of Maralinga village, approximately 800 km NW of Adelaide in southern Australia (Fig. S1). Taranaki was utilized for both major and minor trials resulting in extensive Pu contamination. Trials consisted of one balloon-supported nuclear detonation and one series of minor trials (Table S1). The minor trials, known as Vixen B, were subcritical tests combining nuclear material, 4 TBq of plutonium in each series, and high explosives that purposely resulted in negligible yield in order to study nuclear materials under conditions of explosive shock. These Vixen B trials dispersed 22.2 kg of Pu [3] along particular directions and finely divided plutonium was carried in the direction of prevailing winds [4]. The largest contamination plume resulted in significant contamination 18 km from ground zero.

Soil sampling and dry extraction of hot particles.

Soil samples were taken in 1984 (28 years after the trials) as part of a study [2] to aid in remediation of the site. More than 20 particles were extracted and analyzed using gamma spectrometry and proton-induced x-ray emission (PIXE) spectroscopy [2,3]. These samples were then archived and stored at the Australian Radiation and Nuclear Safety Agency (ARPANSA) at their Yallambie

(Victoria) facility. The soil samples were stored in plastic ziploc bags, double sealed with tape and packed into and sealed in a 55-gallon drum. These were stored in an air conditioned, temperature-controlled basement. There was no moisture present in the sample bag when it was retrieved. The separated particles (Bruce and Potatohead) were sealed with glue between two glass slides that have a central cavity, placed in plastic ziploc bags and stored in a separate sealed 55-gallon drum and stored in the same temperature-controlled basement. Again, no moisture was present in the slide cavity when the samples were retrieved.

We selected one proximal soil sample from the archived samples using gamma spectrometry to screen for the presence of hot particles. A high chance of the presence of hot particles was signified where overall gamma activity was greater than 100 kBq. Hot particles from small aliquots (0.5 g) of the sample obtained from a 3 m wide strip located 440 m north of Taranaki ground zero. In contrast to Ikeda-Ohno [5], who used heavy liquids to extract their particles and centrifugation to extract their particle, we aimed to minimize chemical and physical damage, and we extracted the particles following the dry extraction procedure developed by Burns, Cooper, Williams, and Johnston (Williams, pers. comm.). Each aliquot was sprinkled over an A4 page of thin cardboard covered in double-sided tape to achieve a single particle thin layer. This layer was then scanned systematically using a handheld RadEye gamma survey meter capable of detecting very low gamma energies (45 keV – 1.3 MeV, with a low detection limit of approximately 0.5 μSv/h), then 'hot spots' were removed with a scalpel. Further selection was assisted by means of an optical microscope and particles of interest were removed and examined using a small 2 cm^2 alpha pancake probe to confirm the presence of alpha emitters Pu, U and Am. Finally, selected particles were mounted on 10 μm kapton micromounts (MiTeGen MicroLoops, Ithaca, NY) using cyanoacrylate-based glue and doubly encapsulated with kapton tape to comply with safety requirements at the Diamond Light Source, UK.

This method of locating and extracting hot particles refined from that utilized by Burns et al. [2] avoids altering the chemistry of the particle during preparation for analysis. Hot particles must be extracted from soil media prior to analysis to ensure interference-free analysis on the synchrotron beamline (see SI section "*Data collection at Diamond*").

The effort of extracting particles under dry conditions was also justified when an attempt to remount Chip with less glue (as glue is difficult and time consuming to cut through with a FIB)

involved dunking Chip into an acetone bath for a few minutes but resulted in Chip breaking into multiple pieces when touched with the micro-mount. Either wetting and/or the acetone resulted in Chip becoming more friable. Ikeda-Ohno et al. [5] noted that the fragment that they had recovered from Taranaki was "highly friable" and easily broke into several pieces. They had used wet handling techniques to extract this Pu-bearing fragment.

This study focuses on two hot particles, *Bruce* (one of 12 GNE particles) and *Potatohead* (one of 9 ZD600 particles) originating from the north-east and north-west plumes, see Fig. S1, which were extracted by Burns et al. [2] and the four additional particles that were extracted from the stored soil samples according to the method described above (Fig. S1): *CeresI* (K480i), *CeresII* (K480ii), *CeresIII* (K480iii) and *Chip* (K480iv). Unfortunately, it was not possible to equate *Bruce* and *Potatohead* to the particle numbers attributed by Burns et al. [2]; *Bruce* is either GNE-1, 4, 7, 8 or 9; and *Potatohead* is either ZD600-1, 5, 6, or 9.

Data collection at Diamond Light Source.

The chemical composition and physical nature of the particles were characterised using µSXRF (synchrotron X-ray microfluorescence), µXANES (X-ray absorption near edge structure), µEXAFS (extended X-ray absorption fine structure), µXRD (X-ray microdiffraction) and µSXRF-tomography (X-ray microfluorescence computed tomography). Data were collected at the I18 beam line at the Diamond Light Source, Oxfordshire, UK. I18 is an undulator beamline with a Si (111) double crystal monochromator with an energy resolution of $\Delta E/E = 1.4 \times 10^{-4}$ at 10 keV. Kirkpatrick-Baez mirrors were used to focus the beam on the sample to a spot size of ~2x2 μm^2. Fluorescence (µSXRF, µXANES, µEXAFS) and µSXRF-tomography data were collected with a four-element Si-Drift detector with 130 eV energy resolution. A sCMOS 2D detector with a pixel size of 26 µm was used to collect diffraction (µXRD) data in transmission. µSXRF maps were collected at an incident photon energy of 18.2 keV; XAS (µXANES and µEXAFS) spectra were collected at both the Pu L_3 (18.057 keV) and U L_3 (17.166 keV) edges; µXRD data were collected at 17 keV and µSXRF-tomography was collected at 18.2 keV. The energy for both U and Pu XAS edges were calibrated using a Zr foil (acquired simultaneously in transmission mode), such that the maximum of the first derivative of the Zr K-edge was at 17.99935 keV.

Radiation damage caused by the synchrotron beam may occur at ambient temperature, the extent of damage being dependent on the photon flux incident on the sample [6], which was

2 x 10^{12} photons per second at I18. To minimize potential chemical changes induced by radiation damage, the time spent on each sample was limited to three to eight minutes per µXANES and less than 15 minutes per µEXAFS analysis. Repeat measurements show small changes within the precision envelope of the measurement for all particles, indicating negligible beam damage during data collection.

SXRF data processing.

SXRF data were processed using PyMCA 5.4.1 [7]; PyMCA enables full spectral fitting which is important when samples contain elements with overlapping X-ray lines. Analyzing Pu at the L_3-edge (fluorescence line $L_{\alpha1}$ 14.282 keV) is susceptible to overlapping peaks from greater concentrations of Zr ($K_{\alpha1}$ 15.775 keV) and Sr ($K_{\alpha1}$ 14.165 keV) in the soil matrix. However, by fitting multiple X-ray transitions per element it is possible to distinguish between elements that have some overlapping X-ray lines. PyMCA can produce full quantification of SXRF maps; however, this was not possible in this case, due to the high X-ray attenuation and highly heterogeneous nature of the sample. Note that the U/Pu ratios are least affected, given the relatively high energy of their $L\alpha_1$ emission lines (13.614 keV vs. 14.282 keV), resulting into transmissions of 40.10% versus 44.4% through 10 µm of solid PuO_2.

XRD data processing.

µXRD data were examined using Eva and Topas [8], which enabled phase identification and unit cell refinements. Full Rietveld phase quantification was not possible because of the small X-ray beam and heterogeneous nature of the hot particles: the Rietveld method is designed for homogeneous powders, i.e. a near-infinite amounts of individual crystals in random (or a statistical distribution of) orientations; however, sampling of most phases in the hot particles did not approach this powder limit. In addition, strong absorption by the sample in transmission geometry also affects the relative intensities of the peaks of different phases depending on their location within the hot particle (e.g., absorption length of 19 µm in PuO_2 at 17 keV); and XRD is only sensitive to the portion of the particle that possesses long range periodic order. Hence, the phase quantifications reported in Table S4 are only semi-quantitative. Phases identification was performed by pattern matching, constrained using the elements/phases recognized from µSXRF and SEM+FIB. Rudimentary Rietveld "fits" were run to exclude phases that demonstrably did not

fit (e.g. unit cell changes > 0.01 Å, sometimes this is not evident simply from pattern matching for phases with significant compositional versatility).

Phases used for XRD analysis were: PuO_2 (ICSD 31726), UO_2-c (ICSD 61636), Al-Fe alloy (COD 1541193), BeO (ICSD 15620), Al-Mg alloy (ICSD 150646), α-Pu (ICSD #43335) and Pu_2O_3 (ICSD 71022).

XANES and EXAFS data processing.

XANES and EXAFS data were processed using Athena and Artemis, part of the HORAE package [9] using FEFF v9 [10]. Pu standards are from Conradson et al. [11,12] and U standards are from Syverson et al. [13] and previously collected spectra of uranyl nitrate. Refinement results are presented in Table S2 and S3.

Standard procedures were applied to fit EXAFS data [6]. S_0^2 was determined by invoking Potatohead as a "PuO_2-UO_2 standard" (as determined from XRD): the number of ligands were fixed while the ΔE_0, bond lengths and EXAFS Debye-Waller factors were refined with S_0^2 values fixed and varied from 1.0 to 0.7 (steps of 0.1). By visually examining the fits and looking at the goodness of fit parameters (gofs), the best fit was with S_0^2=0.7. A value of 0.8 would also have been suitable, however S_0^2=1.0 had a statistically significant larger gofs and the plotted fit was inferior to those with lower S_0^2 values. Thus, S_0^2 was fixed at 0.7 for all fits. Comparing the refinements using S_0^2=1.0 and S_0^2=0.7: ΔE_0 varied within the error listed in Table S2, the bond lengths were 0.02 Å longer with S_0^2=1.0, and the EXAFS Debye-Waller factors about twice as large (e.g. σ^2(Pu-O) = 0.016(2) Å^2 with S_0^2=1.0 and 0.008(1) Å^2 with S_0^2=0.7).

Phases used as starting structure for EXAFS were: PuC (ICSD 77305), Pu_2C_3 (ICSD 16510), PuO_2 (ICSD 31726), $PuFe_2$ (ICSD 103608) and δ-Pu (ICSD 43708).

Simulation of XANES spectra.

Given the dearth of XANES spectra that could be found for Pu-C complexes, XANES spectra were simulated (Fig. S4) from *first principles* using the FDMNES package [14-16], following the procedure outlined in our previous studies [13,17,18]. The final states and resulting absorption cross-sections were calculated across the ionization edge using the Finite Difference Method (FDM) to solve the Schrödinger equation. This method allows a totally free potential shape, thus avoiding

the limitations imposed by the Muffin Tin (MT) approximation. The calculations are based on fully relativistic density functional theory (DFT) with local spin density approximation.

Fluorescence tomography data processing.

µSXRF-tomography data were processed utilizing Savu, a python framework developed at Diamond Light Source [19]. Data were further processed using Fiji [20] and Avizo [21].

FIB-SEM.

Particles were sliced using a FEI Helios G4 UX cryo-FIB-SEM at the Ramaciotti Centre for Cryo-Electron Microscopy at Monash University, Victoria, Australia. The FIB-SEM is equipped with secondary electron (SE), back-scattered electron (BSE), energy dispersive X-ray spectroscopy EDS (Oxford, X-Max80 Silicon drift) detector, a through lens detector (TLD) and an in-column mirror detector. Milling was performed using a gallium ion beam, set to the energy of 30 kV and currents between 65 and 1 nA for rough and final polishing steps. The angle of the particle was varied with respect to the incident ion beam (also known as rocking milling) to reduce the appearance of streaks (curtaining effect) on the milled sample face due to a high degree of heterogeneity of the specimen. The imaging was done using an electron beam set to an energy of 3-30 kV, depending on the area of interest. Most of the SEM images were acquired using the BSE detectors as they provide higher compositional contrast but at the cost of lower special resolution, when compared to SE detectors. The currents used during SEM imaging were varied between 200 pA and 3.2 nA depending on the field of view, details required and observed surface charging effects. Electron beam parameters were also varied for EDS acquisition in the same range as for SEM imaging. Lower energy and current beam settings were chosen to investigate low atomic number materials and perform higher accuracy line-scans; whereas higher energy and current were used to look for higher atomic number elements and perform large area EDS overview scans, known as mapping. The AZtec software from Oxford was used to control the detector and collect the EDS data.

Hot particle composition.

Potatohead: single Pu form.

µXRD (Fig. S5, Table S4) indicated the predominance of the isomorphic crystalline phases $(Pu,U)O_{2\pm x}$ and $(U,Pu)O_{2\pm x}$ in *Potatohead*; the asymmetry of the diffraction peaks suggests the presence of two distinct isostructural phases (one Pu-rich, the other U-rich), rather than a single mixed composition (Fig. S5B). The lattice parameters of the two phases were refined to be 5.448(2) Å and 5.418(3) Å using Topas; these values lie between those of the pure endmember UO_2 (5.466 Å) [22] and PuO_2 (a=5.397 Å) [23]. The relatively large errors in the measured unit cells, derived from the average of 7 independent measurements and unit cell refinements on different parts of *Potatohead*, reflect the difficulty in obtaining precise values due to the proximity of the two $(U/Pu)O_{2\pm x}$ phases. The refined unit cells are consistent with significant substitution of U and Pu in each endmember. Plutonium is present mainly as Pu(IV) throughout *Potatohead* (Figs. S4a,c; S5a,b). The bond lengths derived from µEXAFS shell-by-shell fitting (Table S2) are typical of those for $PuO_{2\pm x}$. µXANES results indicate that U is present mainly as U(IV), consistent with $(U,Pu)O_{2\pm x}$ phase. EXAFS data in general are consistent with $UO_{2\pm x}$ (i.e., mostly U(IV)). µSXRF data indicate the presence of a U-rich, idiomorphic grain in Potatohead (Figs. 1, S2). The tomography data (Fig. 1a; Movie S1) show that this grain is actually located on the outside of the *Potatohead* particle. A µXANES spectrum collected from this U-hot spot consisted of ~70% U(VI) and ~30% U(IV) (Fig. S4b,c), and this enrichment in U(VI) is confirmed by the µEXAFS data, showing the presence of uranyl ions (U=O distances = 1.70(2) to 1.77(1) Å; Table S3; Fig. S5b).

Bruce: various forms of Pu.

The Pu µEXAFS spectrum #506 (Table S2; Fig. S4) can be fitted with ~4.9 x O (Pu-O bond length = 2.37(1) Å) + 2 x Pu (Pu-Pu bond length = 3.74(5) Å), consistent with a structurally disordered $PuO_{2\pm x}$. An extra peak in R-space (phase un-uncorrected) is located at ~2.8 Å (Fig. S4A). This peak is not accounted for by adding small amounts of Pu_2O_3 into the fit, but can be fitted with a range of metals (Al, Fe, Ga, Pu) with bond lengths varying from 2.96 to 3.36 Å, suggesting the presence of some Pu-Ga/Fe/Al alloy. δ-Pu was used in the Vixen-B trials (Williams, pers. comm.), and Fe and Al are present in large quantities (µSXRF, FIB-SEM). Pu-Ga alloys exist with bond lengths ranging from 3.01 Å [24] to 3.20 Å [25]; the Pu-Fe system has Pu-Fe bond at 2.98 Å and Pu-Pu bonds at 3.11 Å [26] and Pu-Al has bond lengths at 3.02 Å ($PuAl_3$) [27] while $PuAl_4$ has bond

lengths ranging from 2.20 to 3.83 Å [28]. Note that small amounts of Ga (~2wt%, beyond the detection limit of the FIB-SEM, especially given contamination via the Ga ion beam used in fibbing) are usually added to Pu metal to stabilize the δ-Pu polymorph during machining.

The small peak to the left of the Pu-O peak is most likely due to analytical noise. Although this peak can be fitted with C, the resulting Pu-C bond length was ~1.74 Å, which is rather short for a Pu(IV) compound, and while compounds with plutonyl groups exist, with Pu-O bonds ~1.77 Å [29,30], there is little evidence in the XANES for such high Pu oxidation states.

A second µEXAFS spectrum (#398) measured at another point on *Bruce* had only one broad peak in R-space. This spectrum was modelled using a single Pu-O distance but with a rather large µEXAFS Debye-Waller term, which suggests a range of Pu-O distances (Table S2).

U µXANES data indicate the presence of both U(IV) and U(VI), but the U µEXAFS spectra were too noisy to fit. Linear combination fitting of the µXANES suggest approximately 50% U(IV) and 50% U(VI) (Fig. 4b,c; Table S3).

FIB-SEM – line scans of Bruce.

The FIB-SEM data of Bruce reveals a wonderfully complex interplay of phases and textures. The imaged areas consist of two different types of domains, interpreted to represent at least two immiscible polymetallic melts of Pu-U-Fe(Al)-rich and Al-oxide-rich compositions. The Pu-U-Fe(Al)-rich domains often form spherical inclusions in the Al-oxide-rich domains (Figs. S7(1,4,5,10); S9; S10).

Each of the domains contains a number of different phases. Three main phases were identified in the Pu-U-Fe(Al)-rich domains:

<u>Phase B</u> is the extremely bright phase that occurs in sub-micron isometric crystals (Figs. S9; S10); it contains Pu-U-C possibly with a little Al, and is interpreted to be a Pu-U carbide phase (spectra 32-38 in Fig. S10). The EDS spectra also show some Fe, however this is most likely due to the background Fe-Al alloy phase, given the beam spread at 24 kV.

The BSE contrast of <u>Phase A</u> is the not quite as bright as Phase B. Phase A forms lath-shaped crystals up to ~10 µm in length (Fig. S9; S12). Compositionally (spectra 29-31, Fig. S10), Phase A also is a Pu-U carbide (Pu-U-C as main constituents), but it contains more Al and possibly minor

amounts of Fe compared to Phase B. Oxygen is locally enriched in Phase A (e.g., labels 'A' and 'F' in Fig. S9), possibly as a result of weathering or contamination from the Al-oxide matrix.

Phase C forms the matrix of the Pu-U-Fe(Al)-rich domains, and consists mainly of Fe and Al; it is interpreted to be a Fe-Al alloy (spectrum 26, Fig. S10). This metallic alloy phase contains minor amounts of Pu and U; this alloyed Pu and U likely contribute to the metallic-like component of the µXANES spectra for Bruce. In some Pu-U-Fe(Al)-'blebs', Phase C appears homogeneous in BSE images (e.g., Fig. S8), but in many instances Phase C consists of different domains with different BSE contrasts due to varying Fe:Al ratios. In some cases, the textures suggest eutectic co-crystallization of two alloys with different Fe:Al ratios (e.g., Fig. 2d; Fig. S7(4,7)), but in other cases (Arrow in Fig. 2d; Fig. 3; Fig. S7(6,8)), they are interpreted as resulting from sub-solidus immiscibility in the complex Fe-Al system (see Fe-Al phase diagram [31]). In the latter case, the phase boundaries are decorated by nano-sized Pu-U-rich inclusions exsolved from the parent Fe-Al alloy (Fig. S7(8)).

Phase D encompasses the Al-oxide rich composition coexisting with the Pu-U-Fe(Al) composition, forming either the matrix for Pu-U-Fe(Al)-'blebs' (Figs. 3, S8-S10) or larger domains (Fig. S7(1,2)). Phase D hosts many nanoparticles of Pu-U ranging in size from <50 nm to ~200 nm (e.g., Figs. 7(8,18)). Plutonium and U in these nanoparticles are most likely in oxide form, accounting for the $PuO_{2\pm x}$ component of the µXANES spectrum.

X-ray fluorescence line profiles in *Bruce* (point C in line8, Fig. S9; points B and C in line4, Fig. 4) show that Phase B (Pu-U-carbide) displays elevated oxygen contents locally; these areas usually have lower Pu-U contents (arrow on line4 on Fig. 4b).

Uranium passivation by carbon was demonstrated by Nelson et al. [32], and a uranium carbide phase (UC) was found in depleted uranium particles from Kuwait [33]. This is particularly interesting in light of the time-dependent FIB-SEM results of *Bruce*: *Bruce* was cut twice with the FIB about 10 months apart, and was exposed to ambient conditions (air) between the two FIB sessions. The images in figure in Fig. S12 show the same area imaged 10 months apart: the features are identical, with no evidence of decomposition or extensive oxidation of Phase A or Phase B the small. The differences in contrast evident between the images Fig. S12a and b are due to constraints on the imaging: in (a), the grain was imaged following a FIB cutting (i.e., no coating), whereas the grain

needed to be Au-coated before reimaging after 10 months; image (b) is through this Au-coating, before any FIB polishing.

CeresI: predominantly Pb with heterogenous distribution of Pu + U.

The µXANES spectra collected on CeresI show only the presence of Pu(IV), despite evidence of Pu-U-C deep within *CeresIII*. This can be attributed to the X-ray penetration depth not being sufficient to interact with the Pu-U-C deep within the particle; the outside of *CeresIII* was shown to be oxidized, containing Pb-oxide and Pu-U-oxides (Fig. S11).

The dominant peak of the Fourier transform of the µEXAFS data could be fitted as O from PuO_2. Peaks between ~3.0 and 3.5 Å could be fitted as some sort of Pu-Pu/Fe/Pb/Ga interaction, however, none of the "conventional" compounds (e.g. PuC, δ-Pu, $PuFe_2$, $PuPb_3$) have XANES consistent with the distinctive $PuO_{2\pm x}$ measured, thus it was difficult to assign phases to the remaining peaks (Fig. S4, Table S2). There is also a shoulder to the left of the Pu-O peak. Given the complexity of the particle, formed at elevated temperatures with excess C, H and N, it is not possible to uniquely identify the ligands responsible for this peak, which may be due to some novel compound. Oxygen, carbon and nitrogen are known to form a variety of high-melting, refractory ceramic compounds with Pu [34]. Recently, shorter U-x (x=C, N, O) have been reported: Fox et al. [35] report a U-C bond of 2.383(6) Å and U-N bonds as short as 2.047(6) Å in their study of uranium catalysts.

Previous studies.

Ikeda-Ohno et al. [5] examined a particle from the Maralinga tests with μSXRF and found that the Pu and U distributions were decoupled. Batuk et al. [36] used μSXRF imaging to show that particles from the McGuire Air Force Base accident have both homogenous and heterogenous Pu+U distribution. Erkisson et al. [37] using μSXRF imaging and μSXRF-tomography found that in the particles collected from the Thule accident the Pu occurs predominantly where the U occurs, however the Pu/U intensity distribution varies. Lind et al. [38] demonstrated with SEM-EDX that the particles that they analysed from both the Thule and Palomares accidents had a homogeneous Pu+U distribution (Table S5).

These studies also characterised nature of Pu in these particles and found Pu to exist predominantly as Pu(IV), with evidence of Pu(III), P(V) and P(VI) as well and U to exist as a mix of U(IV) and U(VI). Lind et al. [39] studied three particles from Thule and found evidence of Pu(III)+Pu(IV) homogeneously distributed with U(IV). In, their later study, Lind et al. [38], examined two particles from Palomares and found a mixture of Pu(III)+Pu(IV)+Pu(V) co-existing with U(IV). Of the six particles from Thule analysed by Eriksson et al. [37], two contained predominantly Pu(IV) and two particles were 33% Pu(IV)+ 67 % Pu(VI), and all were mixed heterogeneously with U(IV). Ikeda-Ohno et al. [5] examined five particles from one fragment from the nuclear test site at Taranaki, Maralinga and found a relatively insoluble $PuO_{2\pm x-y}(OH)_{2y}\cdot zH_2O$ phase coexisting with Pb surrounded by an external coating of Ca, Fe and U believed to be acquired from the soil. In (one of) the most comprehensive studies to date, Batuk et al.[36] characterised numerous hot particles and bulk soil & concrete samples from six different sites: Rocky Flats, McGuire Airforce Base, Chernobyl, Mayak, Los Alamos TA-21 and Hanford. Batuk et al.[36] showed the incredibly complexity and variation of these particles, with U varying from UO to U_2O_3 to U(UI) and Pu existing as both well ordered PuO and novel PuO type compounds incorporating additional elements; they concluded that the speciation may, but also may not, be associated with the source terms and histories of the samples.

It is interesting to note that glassy aerodynamic fallout particles from near surface nuclear tests also display chemical and textural heterogeneity[40-43] (Table S5).

Supplementary Figures.

Figure S1. Geographical location of Maralinga.

(a) Location in Australia. (b) Location of major and minor trial sites. (c) Location of studied hot particles within contamination plume contours, with indication of the most likely tests contributing to individual plumes according to Burns et al. (1990) [2].

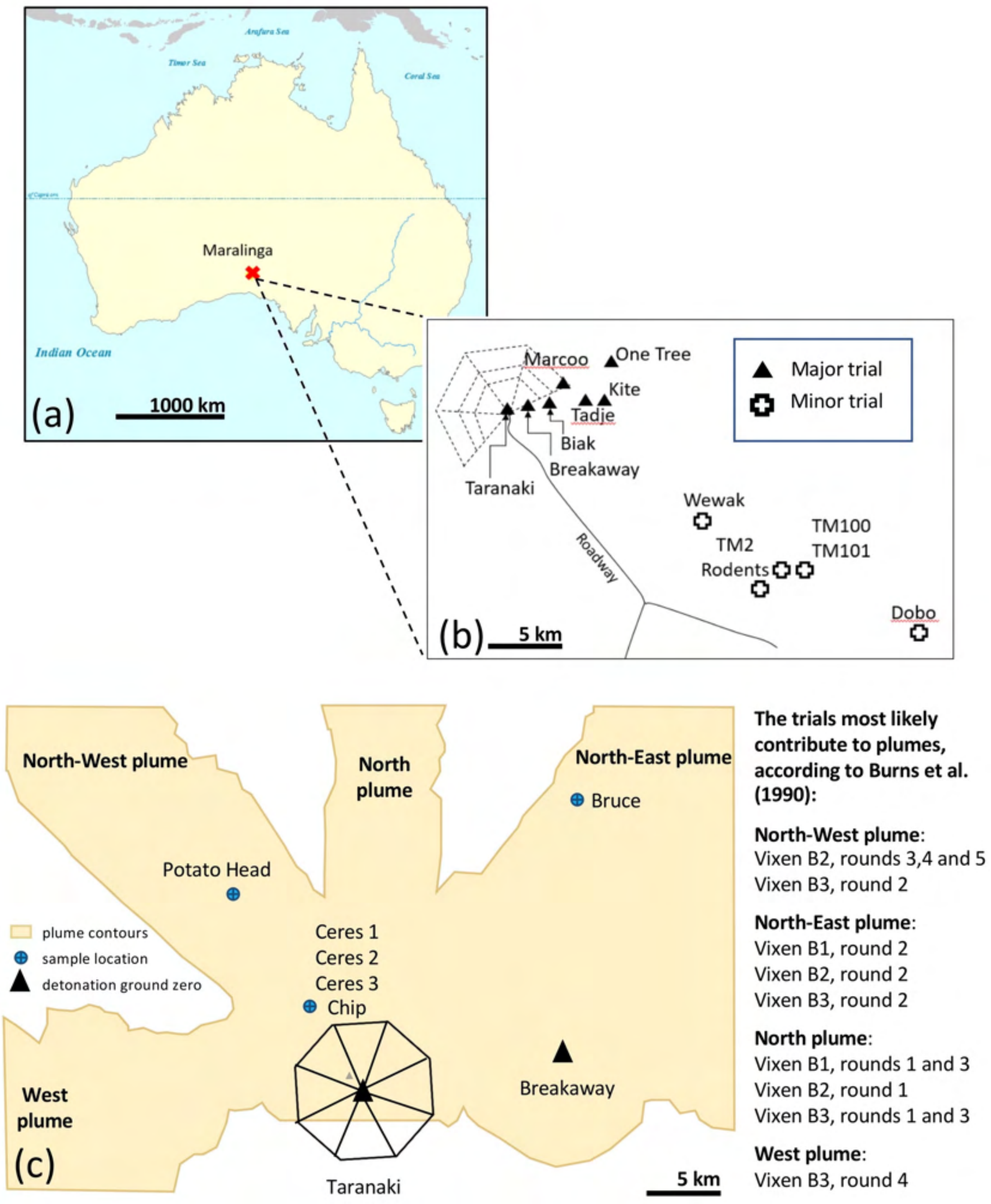

Figure S2. µSXRF characterisation of hot particles from Maralinga.

(a-f) Elemental maps of the six grains, based on region of interest (ROI) and U/Pu intensity ratio maps for *Potatohead* and *Bruce*. *Potatohead* shows U enrichment, relative to Pu, along the rim (a). (g) Ratios of PuLα versus ULα counts for the individual grains; two Pu:U ratios (1.8 and 0.7) are shown for reference. The blue oval in (g) corresponds to the U-rich grain highlighted in the U-map in (a).

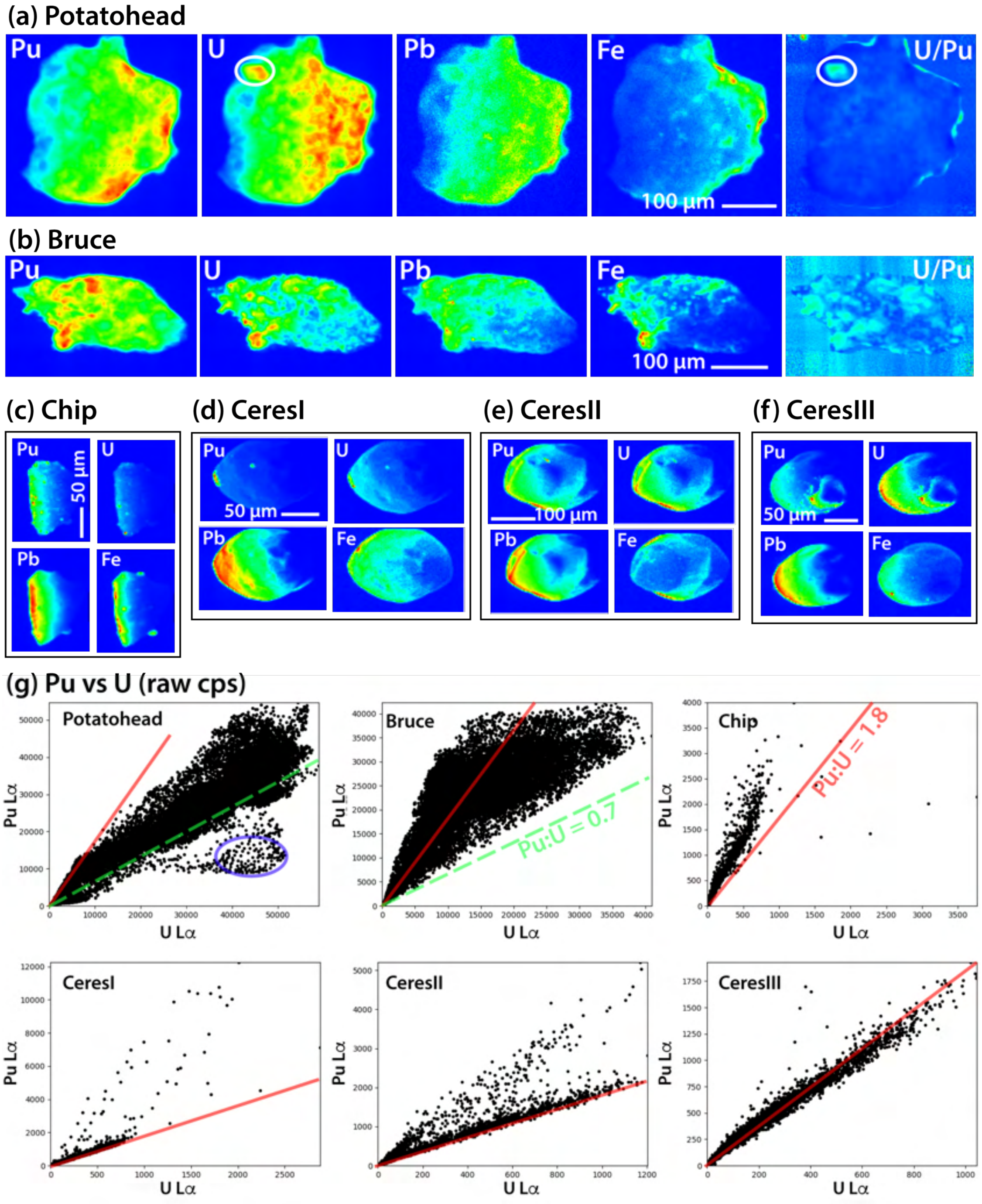

Figure S3. μSXRF spectra of the six hot particles.

The spectra are integrated from the μSXRF maps shown in Fig. S2, and qualitatively highlight the similarity of *CeresI*, *CeresII*, *CeresIII* and *Chip*, and the difference of *Bruce* and *Potatohead*.

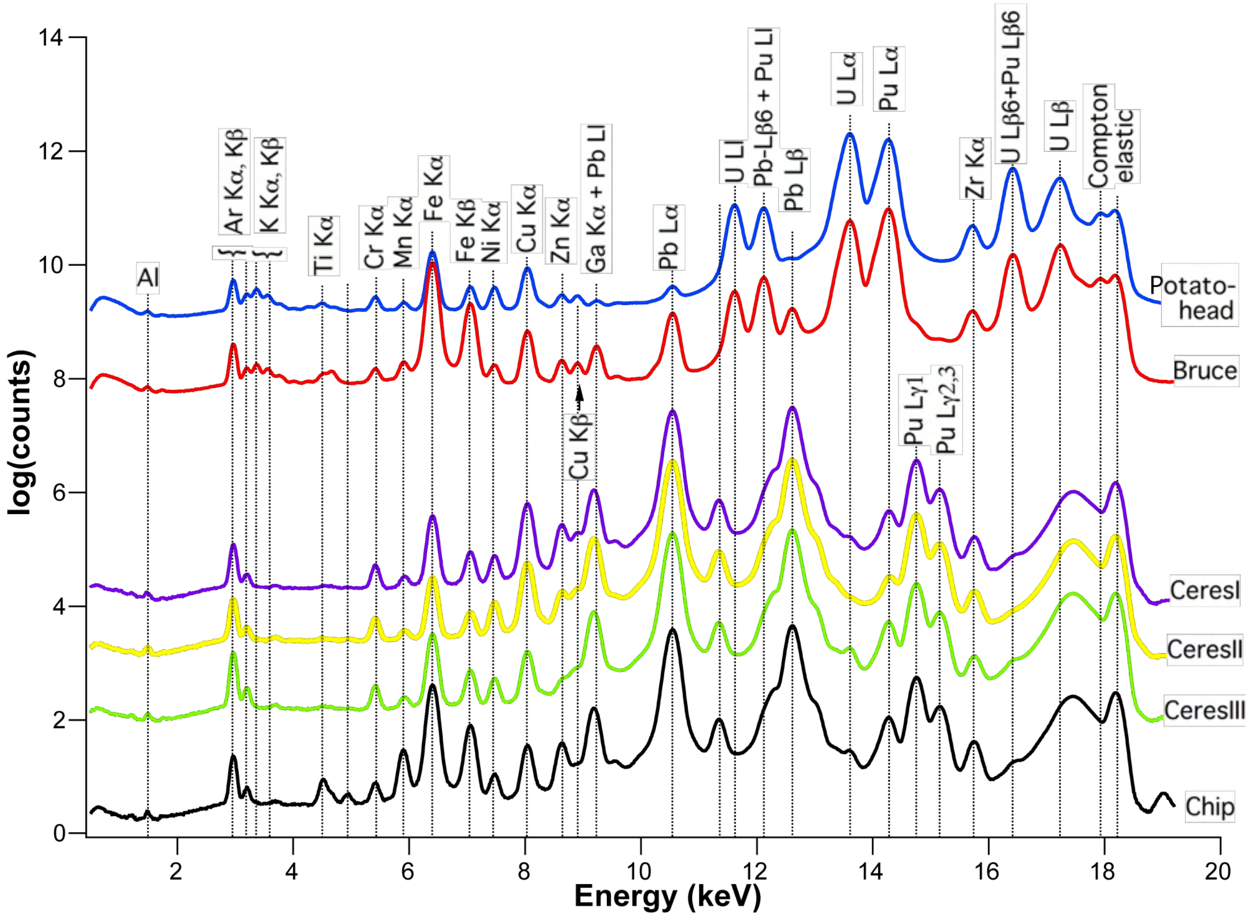

Figure S4. Results of µEXAFS analysis at Pu and U L3edges.

a) Fourier transform of Pu µEXAFS, b) Fourier transform of U µEXAFS, and c) comparison of Pu and U µEXAFS in K-space.

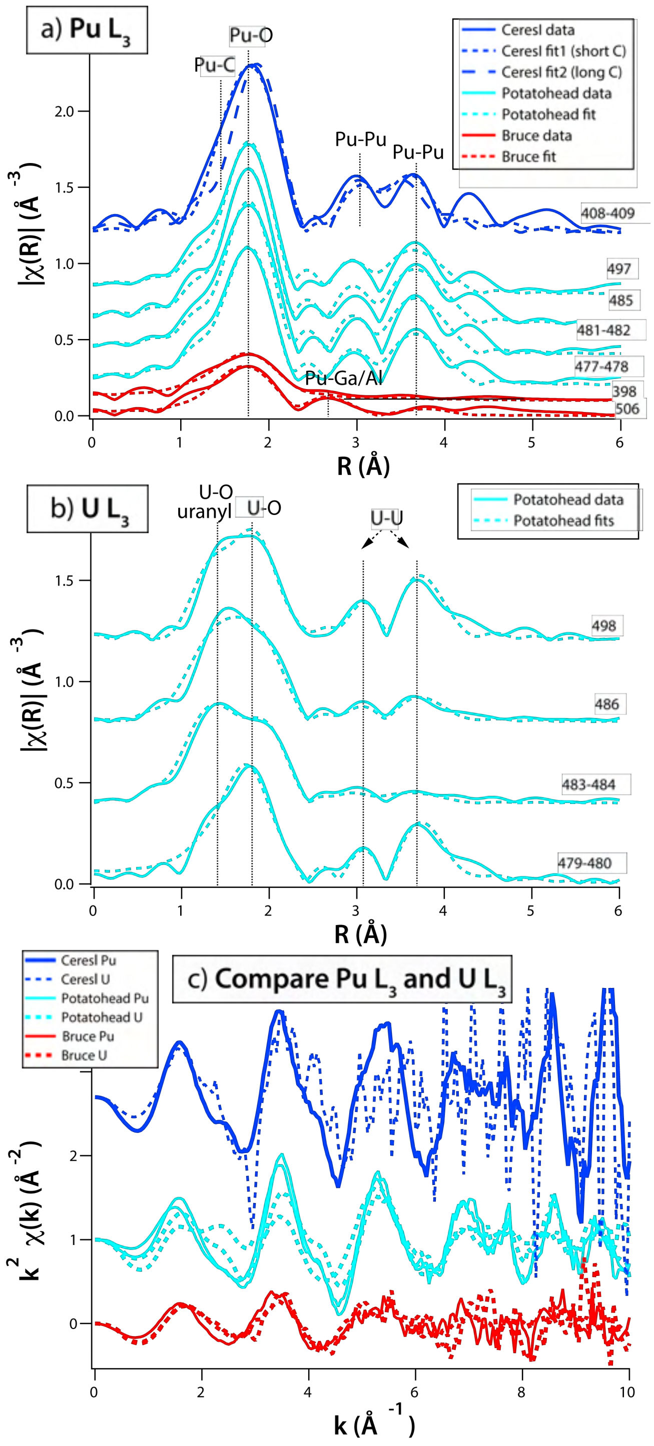

Figure S5. µXRD results for *Potatohead* (data shown as solid lines, fits as dashed lines).

(a) The predominant phases for all diffractograms (taken at different points) are PuO_2+UO_2. A few minor (unidentified) phases are also present. (b) Detail of two diffractograms – showing splitting of peaks suggestive of both PuO_2 and UO_2 phases, with different ratios of these phases across the hot particle. The phases used to fit the *Pototahead* patterns are highlighted in SI Section “*Materials and Methods-XRD data processing*”.

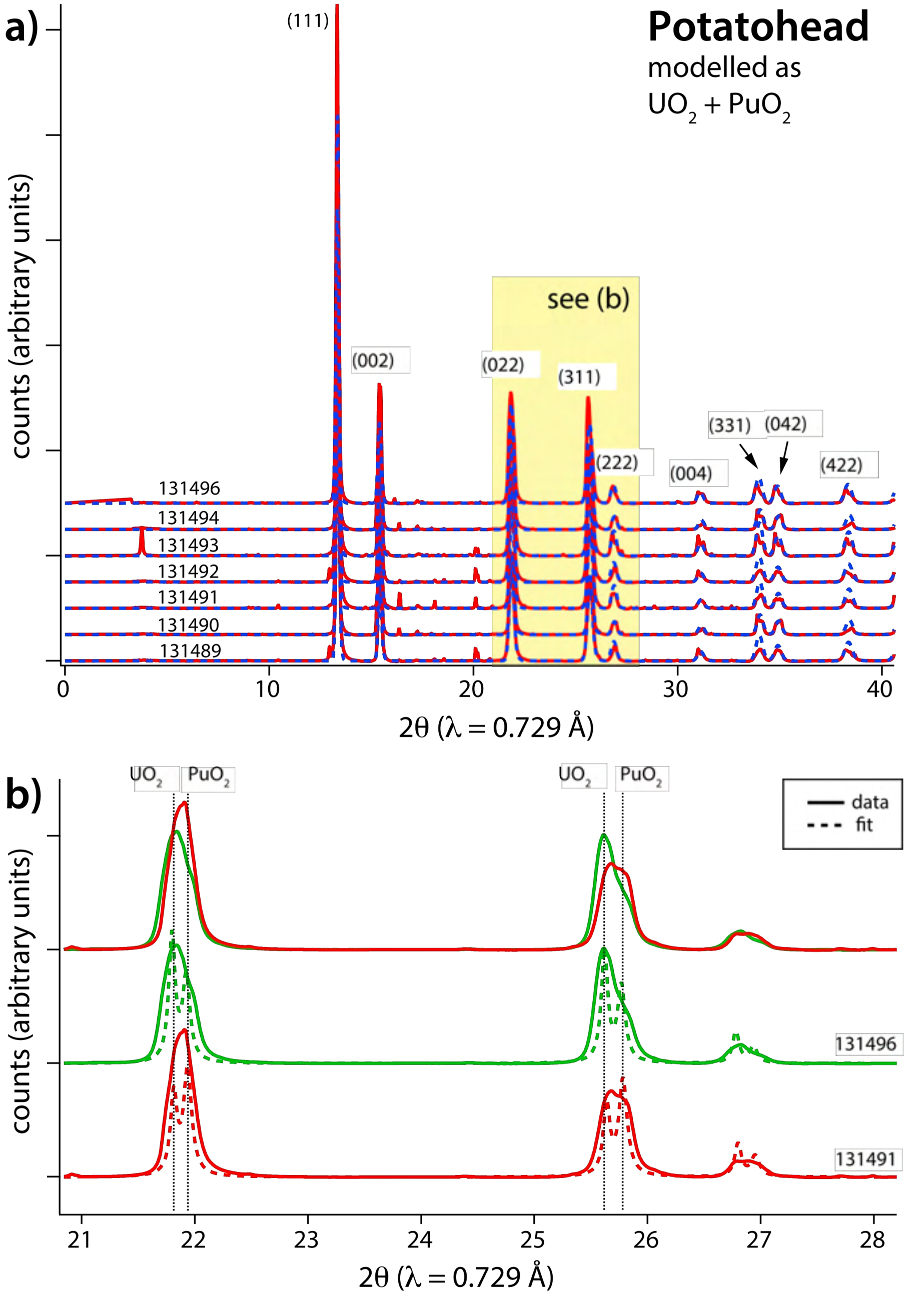

Figure S6. µXRD patterns of *Bruce*.

The diffractograms are from different points on the particle, demonstrating heterogeneity of *Bruce*. The phases used to pattern match *Bruce* are highlighted in SI section "*Materials and Methods-XRD data processing*".

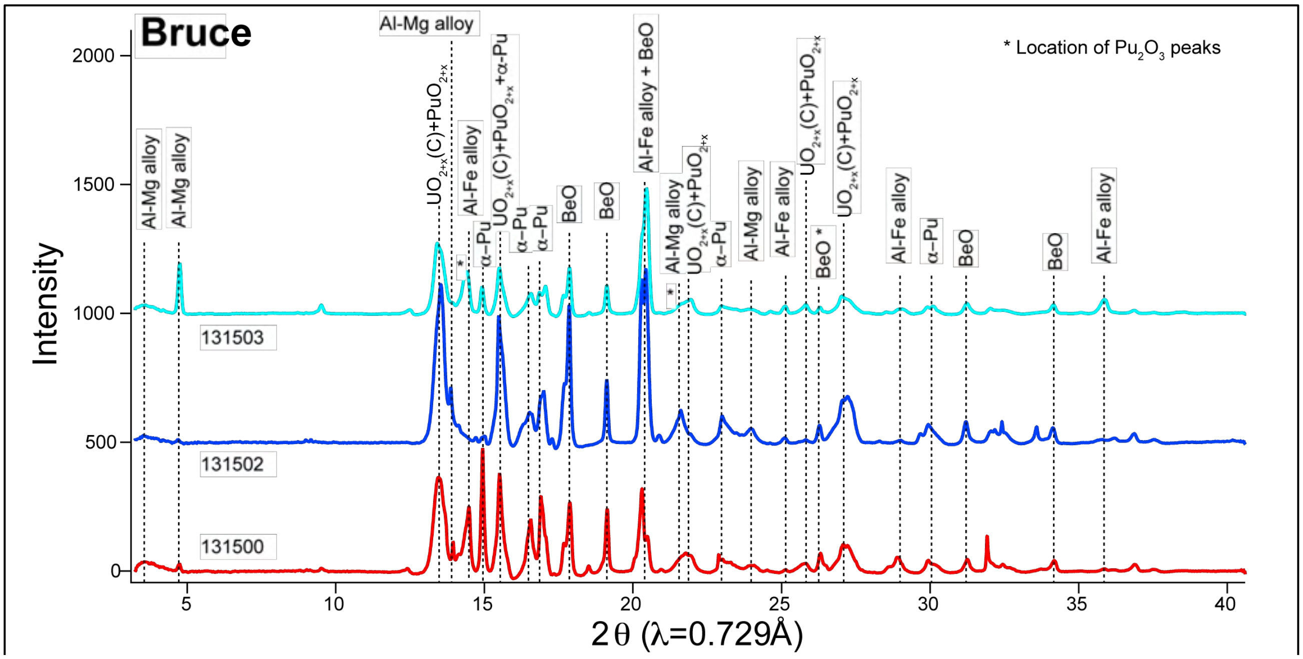

Figure S7. FIB-SEM images of *Bruce* (1 to 21), *Potatohead* (22-27) and *CeresIII* (28-42).

Bruce shows substantial complexity, with regions of high Fe (light grey) and dark grey regions of Al-oxide. Micro- to nano-particles of different Pu-U phases (ranging in size from <50 nm to ~200 nm) are scattered throughout both high Fe and high Al areas (e.g., 12).

Potatohead shows a relatively homogenous mix of Pu-U in an Al matrix.

CeresIII is mainly a grain of metallic Pb with a Pb-oxide rim, covered with a layer rich in Ca-carbonate and Si (potentially a clay mineral). There are small particles of Pu scattered throughout; within metallic Pb they consist of Pb-U-carbide (see Fig. 5), whereas in the Pb-O rim, they are Pu-U-oxides. Fine cracks near Pu-U-rich grains within fresh metallic Pb (32) are suggestive of radiation damage (Frenkel pair alpha decay).

The different coloured borders highlight zoomed regions of the region shown in the top-left image within the highlighted group of images.

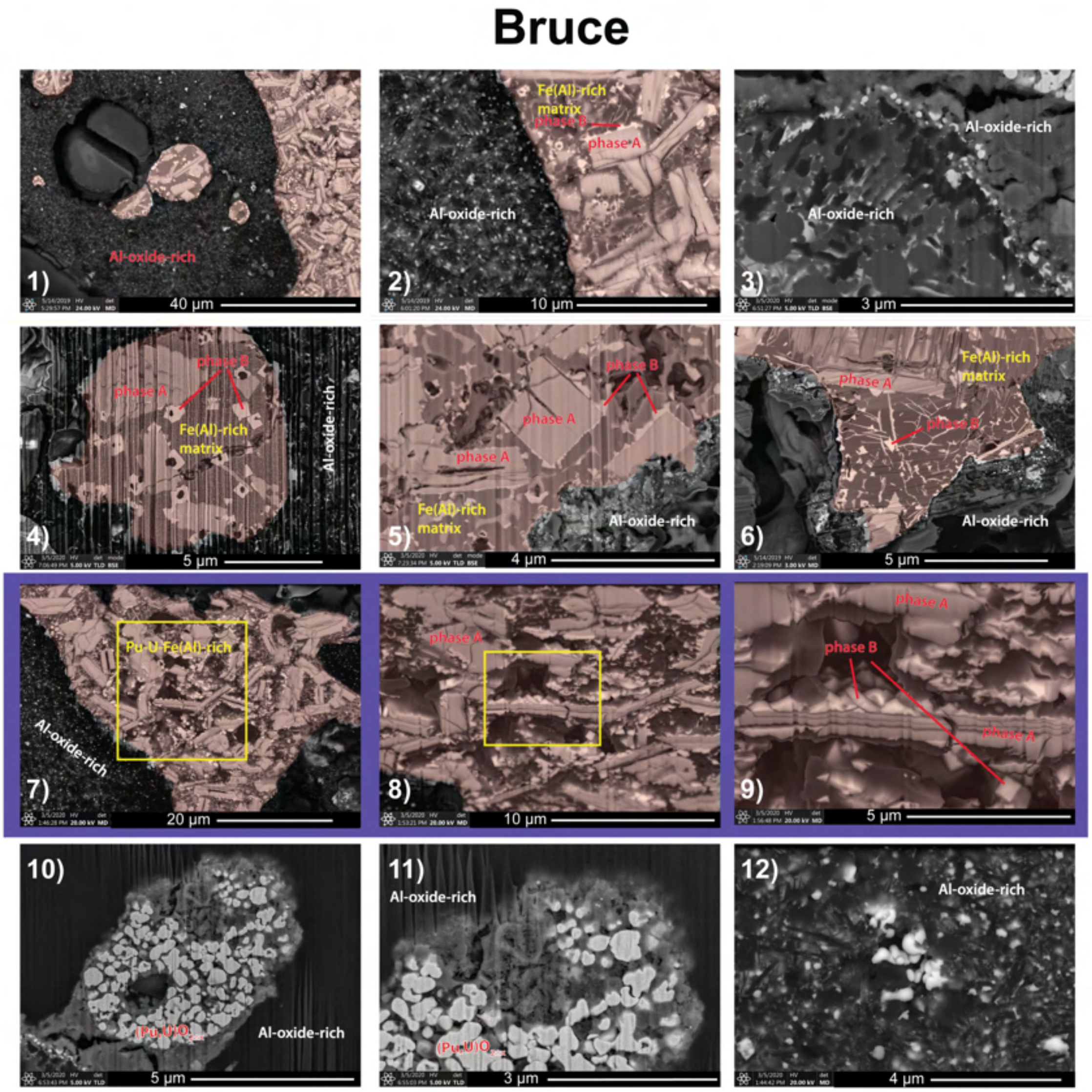

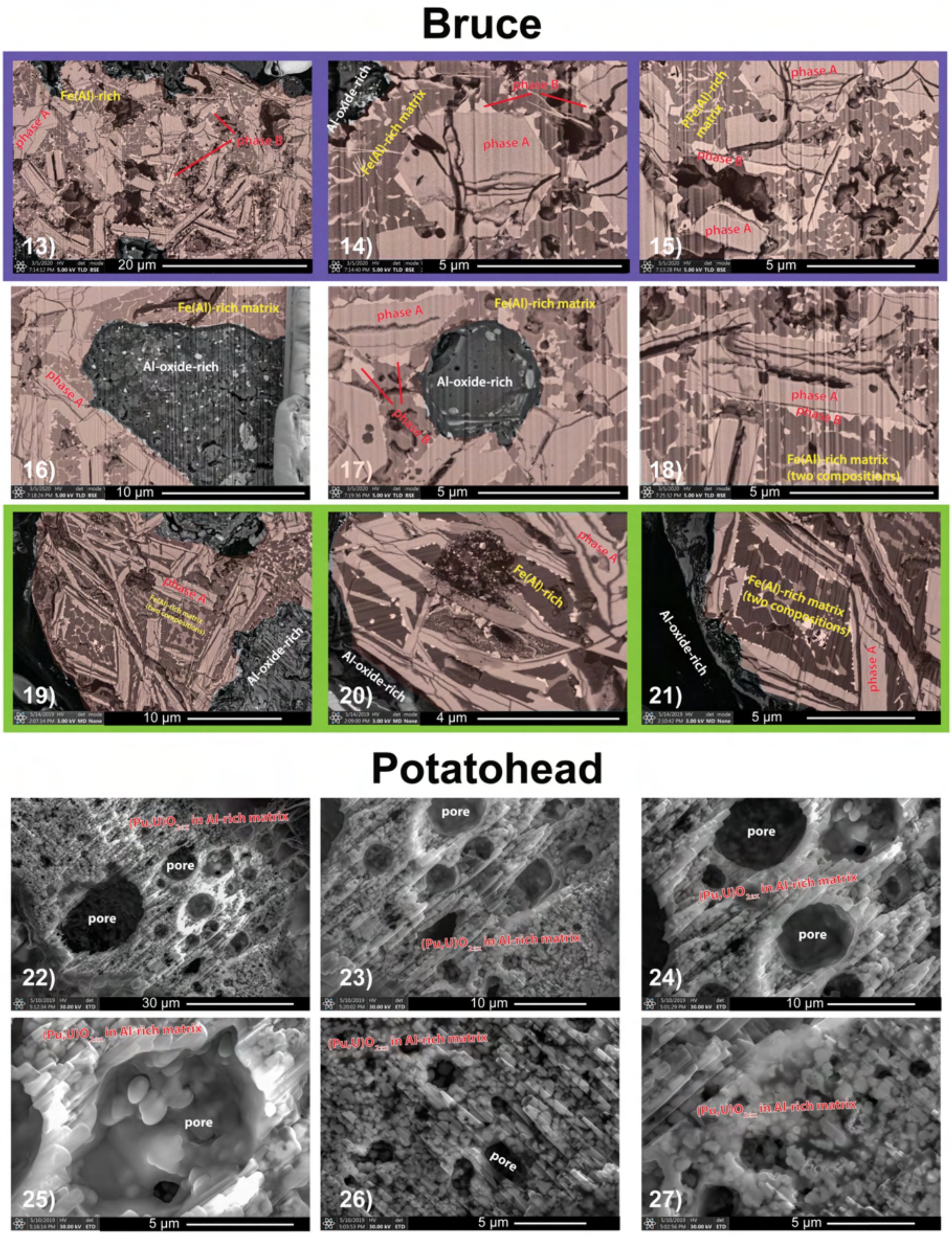
Bruce
Fe(Al)-rich
phase A
phase B
13)
20 µm
Al-oxide-rich
Fe(Al)-rich matrix
phase A
phase B
14)
5 µm
phase A
Fe(Al)-rich matrix
phase B
phase A
15)
5 µm
Fe(Al)-rich matrix
Al-oxide-rich
phase A
16)
10 µm
phase A
Fe(Al)-rich matrix
Al-oxide-rich
phase B
17)
5 µm
phase A
phase B
Fe(Al)-rich matrix
(two compositions)
18)
5 µm
phase A
Al-oxide-rich
19)
10 µm
phase A
Fe(Al)-rich
Al-oxide-rich
20)
4 µm
Fe(Al)-rich matrix
(two compositions)
Al-oxide-rich
phase A
21)
5 µm
Potatohead
(Pu,U)O2±x in Al-rich matrix
pore
pore
22)
30 µm
pore
(Pu,U)O2±x in Al-rich matrix
23)
10 µm
pore
(Pu,U)O2±x in Al-rich matrix
pore
24)
10 µm
(Pu,U)O2±x in Al-rich matrix
pore
25)
5 µm
(Pu,U)O2±x in Al-rich matrix
pore
26)
5 µm
(Pu,U)O2±x in Al-rich matrix
27)
5 µm

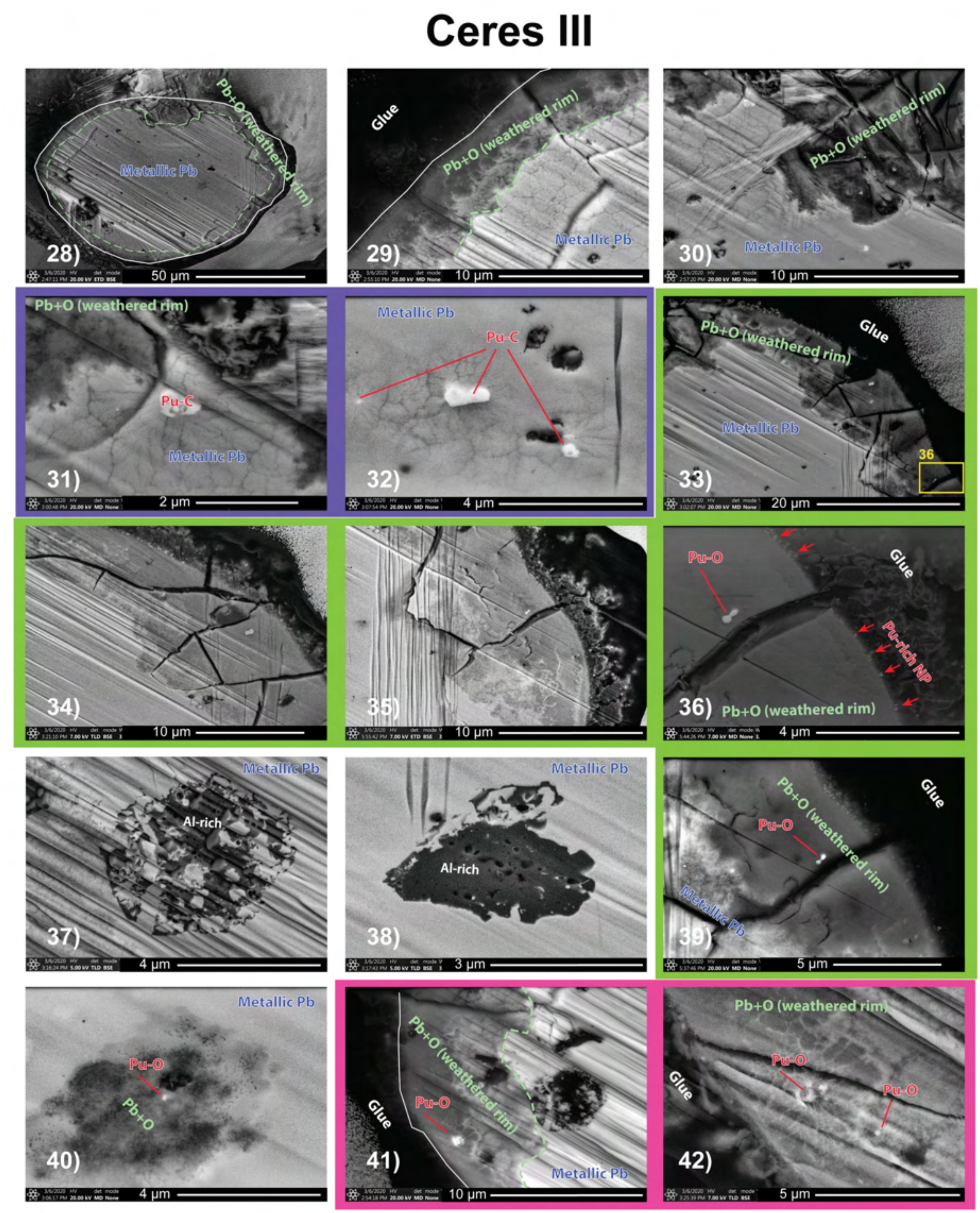
Ceres III
28)
Metallic Pb
Pb+O (weathered rim)
50 µm
29)
Glue
Pb+O (weathered rim)
Metallic Pb
10 µm
30)
Pb+O (weathered rim)
Metallic Pb
10 µm
31)
Pb+O (weathered rim)
Pu-C
Metallic Pb
2 µm
32)
Metallic Pb
Pu-C
4 µm
33)
Pb+O (weathered rim)
Glue
Metallic Pb
36
20 µm
34)
10 µm
35)
10 µm
36)
Pu-O
Glue
Pu-rich NP
Pb+O (weathered rim)
4 µm
37)
Metallic Pb
Al-rich
4 µm
38)
Metallic Pb
Al-rich
3 µm
39)
Pb+O (weathered rim)
Pu-O
Glue
Metallic Pb
5 µm
40)
Metallic Pb
Pu-O
Pb+O
4 µm
41)
Pb+O (weathered rim)
Pu-O
Glue
Metallic Pb
10 µm
42)
Pb+O (weathered rim)
Pu-O
Pu-O
Glue
5 µm

Figure S8. FIB-EDX elemental maps of *Potatohead* and *Bruce*, collected at 24 kV.

(a,b,d) EDS elemental maps highlighting the co-location of Pu-U-Al at the µm- to sub-µm-scales in *Potatohead*, with minor amounts of Fe (c) that do not necessarily correspond with the location of Pu-U-Al. The SEM image of this area is shown in (e).

In contrast, in *Bruce*, there are distinct Pu-U-Fe-Al-rich and Al-oxide-rich regions (f-i). These areas as also labelled in the SEM image in (j).

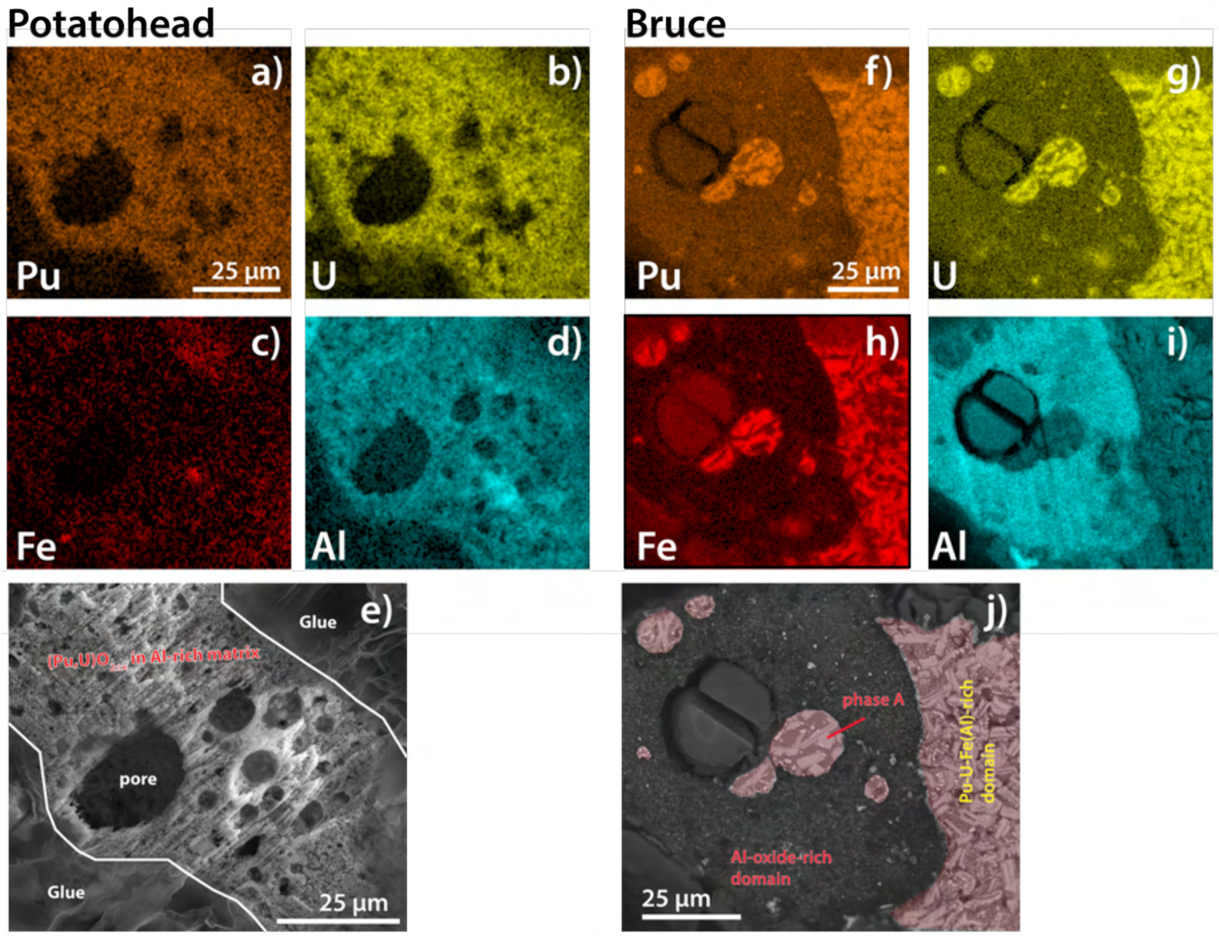

Figure S9. FIB-SEM EDX line scans across a Pu-U-Fe rich ‘blob’ within *Bruce*. Data were collected at 20 kV acceleration voltage.

The lath-shaped crystals seen in (c) (labelled ‘B’, ‘C’, ‘G’; Phase A) are a Pu-U-carbide with minor Al±Fe. The brightest area (‘H’; Phase B) contains relatively more U and less Al than Phase A.

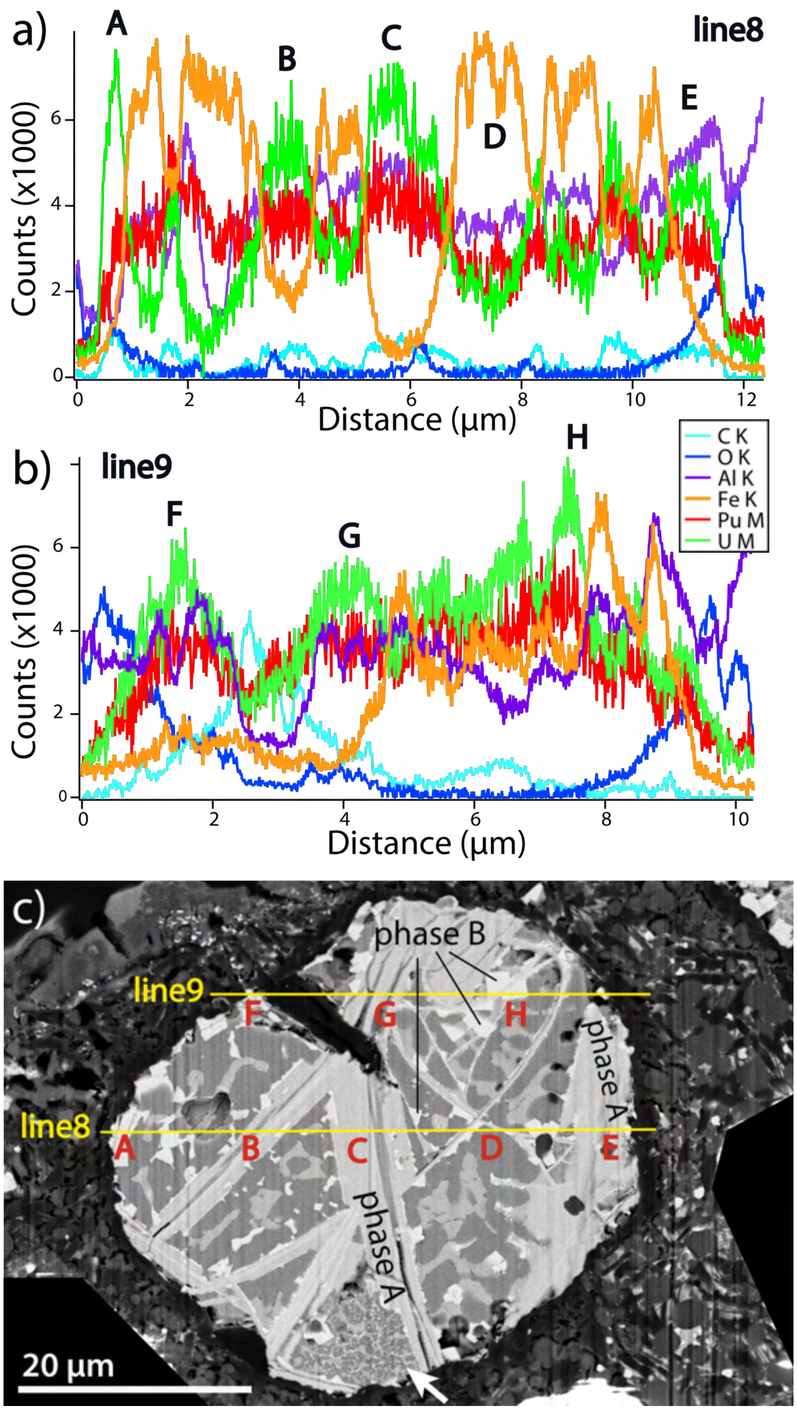

Figure S10. FIB-SEM point spectra analysis within *Bruce*.

The in-column mirror detector image (a) was taken at 20 kV acceleration voltage, but the point spectra shown in (b) were measured at 5 kV acceleration voltage. Counts in Spectra 3 and 4 had to be multiplied by 100 to be comparable with the other two spectra. The bright area consists of Pu-U-C (Spectrum 1; Pu-U X-ray lines are not excited at 5 keV, but higher kV data (not shown) demonstrated the presence of Pu-U), the light grey area (Spectrum 2) joining the Pu-U-C particles is dominated by Fe-Al; and the dark matrix is predominantly Al with some N and O (spectrum 3) and Al-oxide (Spectrum 4).

(c) The darkest areas (dark blue points) are predominantly an Al-oxide matrix (d), containing sub-µm-sized Pu-U-Fe-C inclusions (most ≤ 400 nm in diameter). The darker grey within the circular area (magenta-coloured point #26) comprises predominantly Fe with Al and some Pu-U. The red points consist of Pu-U-C-Al (Phase A) with some Fe. The brightest phase indicated by green points (Phase B) consists of Pu-U-Al-C with Fe; it should be noted that, given the size of these domains and the incident electron beam acceleration voltage of 24 kV, some (or all) of the Fe signal may be from the surrounding background.

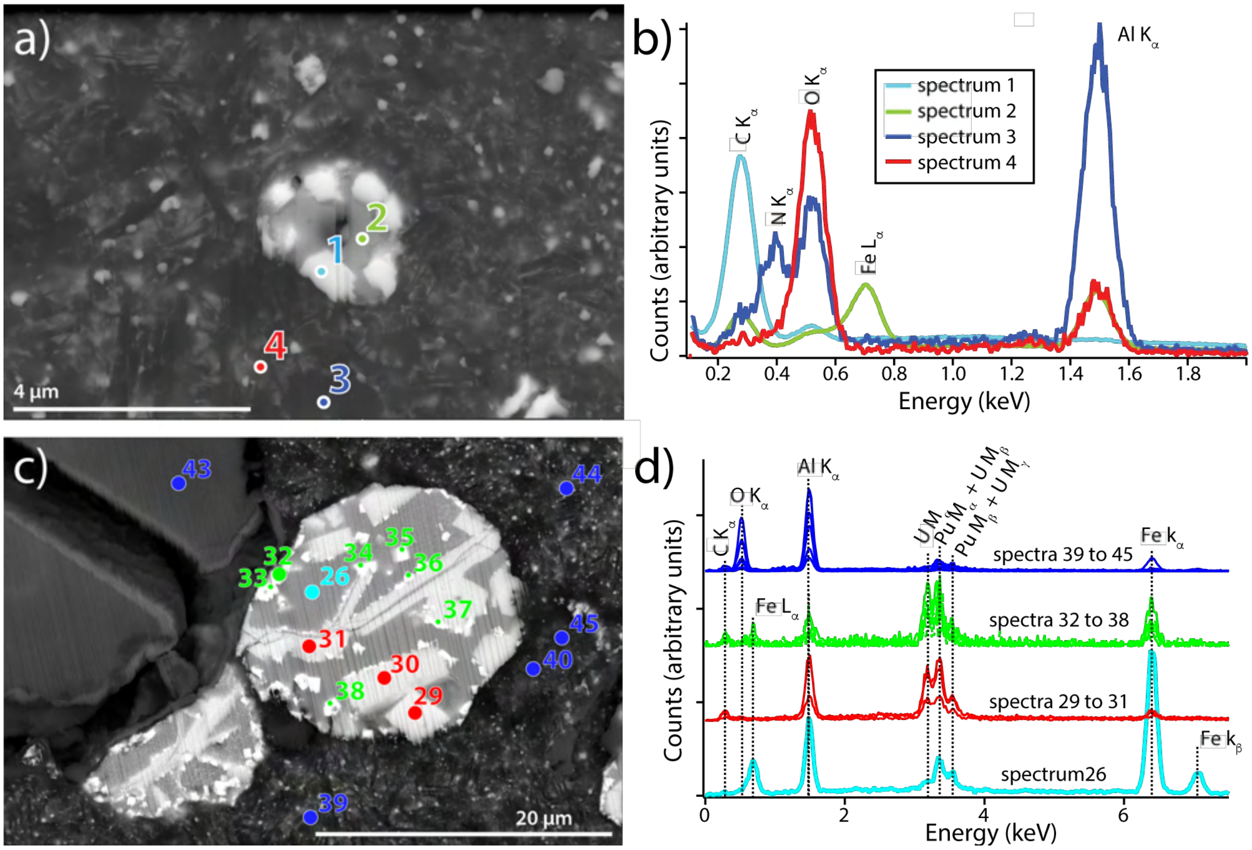

Figure S11. Composition and texture of the *CeresI* hot particle.

(a) Secondary electron image of FIB-SEM cut through *CeresI*. (b-d) EDS element maps of Pb, Cu and Fe of the top image. The texture is consistent with formation via cooling of a melt. Note: the cut was challenging because of the large amount of protective glue surrounding the grain. Poor ion polish resulting for the thick layer of glue used to protect this particle for synchrotron experiments is responsible for the textured surface.

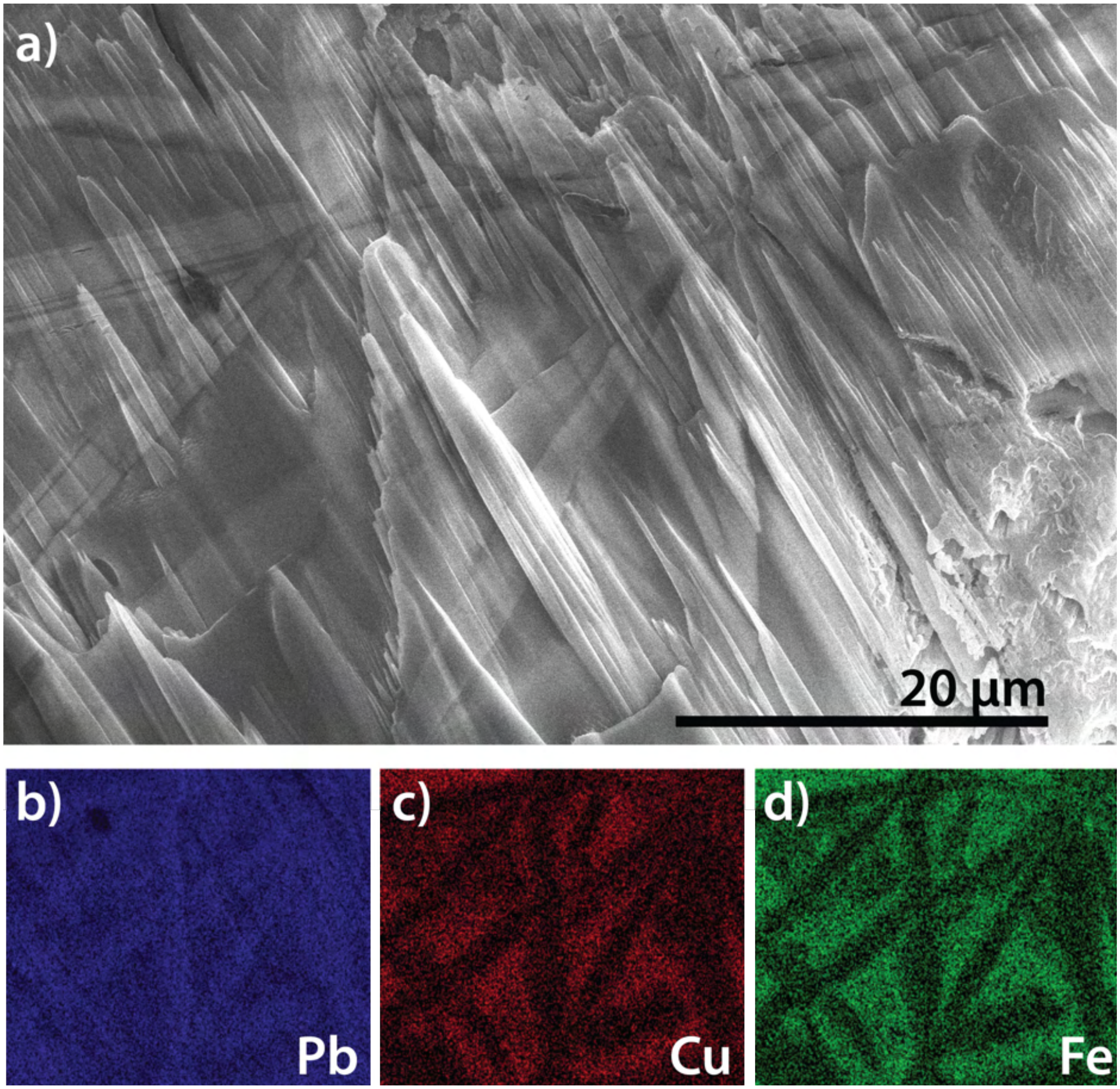

Figure S12. FIB-SEM images of *Bruce* taken ~10 months apart, showing relative stability of the contained phases upon exposure to air.

(a) In-column mirror detector image of ion-polished surface taken on 14 May 2019, and (b) Lens Detector image or the same area taken on 5 March 2020. Bruce was Au-coated immediately prior to the 5 March session, but image (b) was taken before ion polishing. The Pu-U rich phases (Phase B, bright cubes; and Phase A, light grey lath-shaped crystals) retained their shape and imaging contrast relative to the background Fe-Al matrix. Overall, the images show no evidence of significant oxidation of the Pu-U rich phases following exposure to atmospheric, ambient conditions for around 10 months.

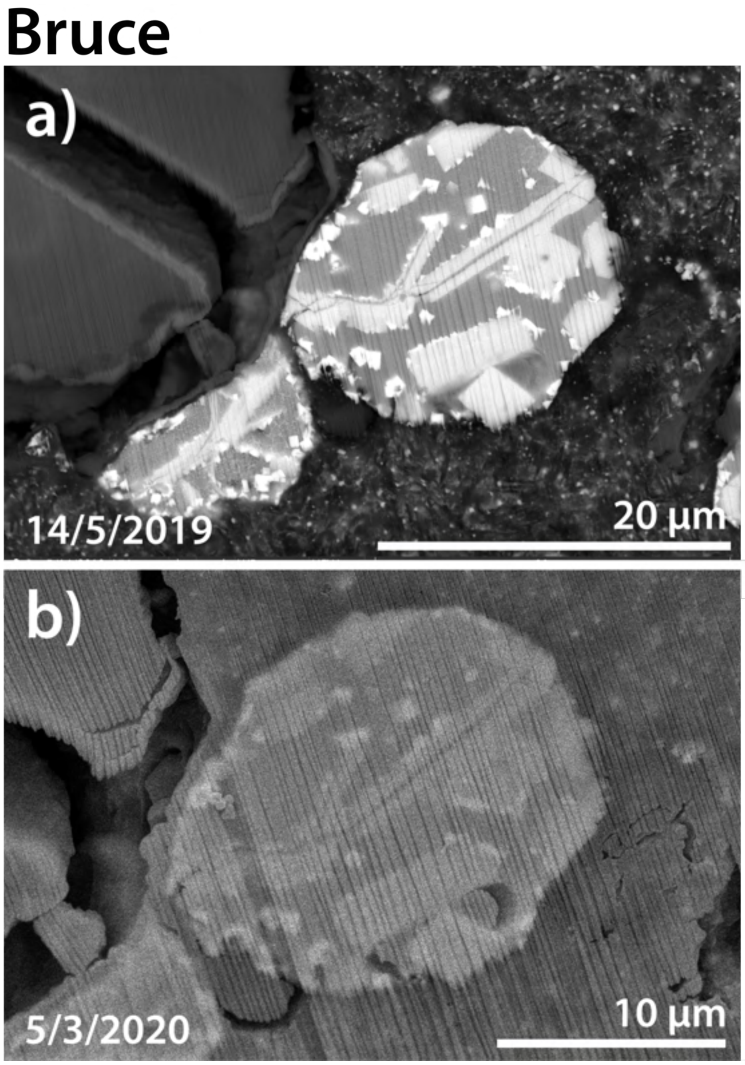

Supplementary Material: Tables.

Table S1: A history of British nuclear tests in mainland Australia. The studied particles originate from the Vixen B series of trials.

Minor Trials						
	Operation Vixen B	Taranaki Area, Maralinga (12)	1960–1963	NA	NA	A total of 12 *Vixen B* Trials were conducted to determine the effects of an accidental detonation of some of the high explosive in the weapon and involved 22 kg of Pu. All of the *Vixen B* rounds were carried out at Taranaki where they produced the worst of the contaminated areas at Maralinga. **The materials used and dispersed in the Vixen B series included ^{239}Pu (22.2 kg), ^{235}U (22.4 kg), ^{238}U (24.9 kg) and beryllium (17.6 kg)**. The weapons grade Pu used during VIXEN B trials was ~95% ^{239}Pu, ~5% ^{240}Pu, and ~0.5% ^{241}Pu. At the time of Operation Brumby, there were 21 pits in the Taranaki area containing about 830 t of material contaminated by 20 kg of plutonium. The remaining plutonium, now estimated to be about 2 kg, was dispersed on the Range.
	Operation Kittens	Naya Area, Maralinga (94) & Emu Fields (5)	1953–1961	NA	NA	A total of 99 trials were conducted on the development and testing of neutron initiators. The initiator consisted of a radioactive substance brought into contact with beryllium by a chemical explosive. In later Kittens trials, the neutron output was used to assess the performance of the high explosive assembly that compresses the fissile material in the core. The experiments at Emu Fields dispersed 36g of beryllium and 15 TBq of ^{210}Po into the surrounding area. The Kittens experiments at Naya Area dispersed 259 TBq of ^{210}Po, 750 g of beryllium and 120 kg of natural and depleted uranium.
	Operation Tims,	Naya and Kuli Areas, Maralinga (321)	1955–1963	NA	NA	The *Tims* experiments were designed to measure the compression of a simulated core of an atomic weapon and the design of the high explosive component to achieve maximum compression. A total of 321 experiments were carried out in the Naya and Kuli areas to investigate uranium and beryllium tampers, and early subcritical hydro-nuclear tests. These experiments used and dispersed beryllium (77 kg), natural uranium ore (825 kg) and ^{238}U (6800 kg). In 1960, twelve Tims experiments carried out at TM100 and TM10l used about 1.2 kg of plutonium.
	Operation Rats	Naya and Dobo Areas, Maralinga (125)	1956–1960	NA	NA	The *Rats* experiments had a similar objective to the Tims experiments but used a different technique to determine the compression. A total of 125 trials of explosive dispersal of uranium were conducted; the materials used included ^{46}Sc (80 TBq), ^{238}U (180kg), ^{210}Po (15 TBq) and ^{212}Pb (4.4 TBq).

	Operation Vixen A	Wewak Area, Maralinga (31)	1959–1961	NA	NA	A total of 31 *Vixen A* trials were conducted to investigate the spread of radioactive and toxic materials that might result from an accidental fire on a nuclear weapon, and involved a total of 0.98 kg Plutonium. Three kinds of experiments were conducted: combustion in controlled petrol fire (800-1200ºC) for Uranium and Beryllium, and 600-1000ºC for Plutonium; combustion in air in an electric furnace operating at 600-800ºC used only for uranium; and dispersion by high explosives consisting of the explosive implosion assemblies from nuclear weapons. The materials used during these trials included 6.0 kg of beryllium of which 4.2 kg was dispersed, 68 kg of natural and depleted uranium, 0.98 kg of plutonium of which 0.58 kg was dispersed, 3.6 TBq of ^{210}Po and 0.07 TBq of ^{227}Ac.
**Test Series**						
	**Test Name**	**Location**	**Date**	**Yield**	**Explosion Conditions**	**Comments**
Totem	Hurricane	Monte Bello (off Trimouille Island) 20.41°S 115.55°E	3rd Oct, 1952	25 kt	Ocean Surface burst (HMS Plym)	A British Blue Danube Design with a levitated pit plutonium implosion device (air gap between the uranium tamper and the plutonium core) was used. The bomb was exploded inside the hull of Plym, anchored 350 m off Trimouille Island. The fallout is unknown, although changes in wind pattern likely led to land contamination.
Totem	Test 1	Emu Field, South Australia 28.68°S 132.34°E	15th Oct 1953	10 kt	Steel Tower, 31m	A British Blue Danube design was used. The main purpose of the trial was to determine the acceptable limit on the amount of ^{240}Pu present in a bomb. Reports highlight a black mist generated from Totem-1 across the landscape at Wallatina and Welbourn Hill stations in Granite Downs, 175 km from the test site, and led to "unacceptably" high levels of radioactive contamination.
Mosaic	Test 2	Emu Field, South Australia 28.70°S 132.35°E	27th Oct 1953	8 kt	Steel Tower, 31m	A modified British Blue Danube design was used. The yield was much bigger than the expected 2–3 kt, however, was below the estimated maximum. High winds dispersed the radioactive cloud so that it had dissipated to the point where it could not be tracked.
Mosaic	G1	Trimouille Island in the Monte Bello Islands, Australia	16th May 1956	15 kt	Aluminium Tower, 31m	The first British test using a boosted fission weapon design with lithium deuteride fusion fuel. The fallout cloud initially moved out to sea as predicted, but then reversed direction and drifted across northern Australia.

		20.23°S 115.55°E				
Buffalo	G2	Alpha Island in the Monte Bello Islands, Australia<br>20.40°S 115.53°E	19th June 1956	98 kt	Aluminum Tower, 31m	A boosted fission weapon design with lithium deuteride fusion fuel and a Uranium tamper was used. It is the largest nuclear device ever detonated in Australia with the highest yield. The bulk of the fallout drifted over the Arafura Sea, but owing to different winds at different altitudes, part of it drifted over the mainland.
Buffalo	One Tree Site, Round 1	Maralinga (One Tree)<br>29.87°S 131.66°E	27th Sept 1956	15 kt	Aluminum Tower, 31m	A Red Beard un-boosted fission implosion weapon with a composite core (containing both weapons-grade plutonium and weapons-grade ^{235}U) was used. Two radioactive fallout clouds resulted from the test with rainfall depositing some fallout in the Brisbane-Lismore area.
Buffalo	Marcoo Site, Round 2	Maralinga (Marcoo)<br>29.88°S 131.62°E	4th Oct 1956	1.5 kt	Ground	A Blue Danube bomb equipped with low yield Mark I enriched uranium core was used. The weapon was lowered into a concrete pit and left a crater 49m wide and 12m deep. The fallout crossed the east coast 25 to 30 h after the detonation.
Buffalo	Kite Site, Round 3	Maralinga (Kite)<br>29.89°S 131.66°E	11th Oct 1956	3 kt	Air drop, 150m	This was Britain's first air drop test. It tested a low-yield Blue Danube core with less fissile material. Two clouds formed; a low-level one at 2,100m that dropped all its radioactive material inside the prohibited area, and a high-level one at 3,700m that deposited negligible amount of fallout over South Australia, Victoria and New South Wales.
Antler	Breakaway, Round 4	Maralinga (Breakaway)<br>29.89°S 131.60°E	22nd Oct 1956	10 kt	Aluminum Tower, 34m	This was the second test of a boosted Red Beard fission implosion weapon with a composite core (containing both weapons-grade plutonium and weapons-grade ^{235}U). The radioactive fallout reached 11,000m but soon became widely dispersed between Darwin and Newcastle.
Antler	Round 1	Maralinga (Tadje)<br>29.89°S 131.65°E	14th Sept 1957	1 kt	Aluminum Tower, 31m	The test of Pixie, a lightweight small diameter implosion device with a plutonium core was conducted using cobalt pellets as a test diagnostic for measuring yield. Later reports found personal handling of these pellets led to exposure of ^{60}Co. The results of the continental sticky paper survey showed that the fallout levels were low; the maximum observed was at Alice Springs, and most of the fallout occurred to the north-east and north-west of Maralinga. Small amounts of fallout, close to the detection limits were detected in Adelaide and Melbourne.

Antler	Round 2	Maralinga (Biak) 29.89°S 131.62°E	25th Sept 1957	6 kt	Aluminium Tower, 31m	A lightweight plutonium implosion device named Indigo Hammer, intended for a surface to air defense missile (Bloodhound) and thermonuclear primary applications was tested. A calculation immediately after firing using the observed height of the cloud predicted that the fallout would exceed out to a distance of about 240 km.
Antler	Round 3	Maralinga (Taranaki) 29.89°S 131.59°E	9th Oct 1957	25 kt	Balloon-burst over land, 300m	

Notes: The data are adapted from Symonds [1] and the Maralinga Rehabilitation Technical Advisory Committee (MARTAC) report [4].

* These minor tests were performed without formal Australian government approval, and without any advice being given to the Australian government by Australian or British scientists.

*1 TBq = 10^{12} Bq

Table S2. Particle Pu-L_3 edge EXAFS refinements.

nO is the number of oxygen atoms coordinated to the actinide of interest. RO is the Pu/U-O distance (Å). σ^2O (Å^2) is the EXAFS Debye-Waller factor; nPu is the number of Pu atoms. RPu is the Pu-Pu distance (Å), σ^2Pu (Å^2) is the EXAFS Debye-Waller factor; similarly for Pb and Ga. Values in parentheses are the errors of the fitted values.

(i) Potatohead data were fitted over the following ranges: $2 \leq k \leq 9.5$ Å^{-1}, $1 \leq R \leq 5$ Å.

**Pu**	*n*O	*R*O (Å)	σ^2O (Å^2)	*n*Pu	*R*Pu (Å)	σ^2Pu (Å^2)	ΔE$_0$ (eV)	$\chi2_{red}$
Potatohead [477-478]	8 (fix)	2.32(1)	0.008(1)	12 (fix)	3.83(2)	0.010(2)	5.4(4)	140
Potatohead [481-482]	8 (fix)	2.32(1)	0.007(1)	12 (fix)	3.83(1)	0.009(1)	5.4(4)	140
Potatohead [485]	8 (fix)	2.32(1)	0.006(1)	12 (fix)	3.83(2)	0.009(2)	5.4(4)	140
Potatohead [497]	8 (fix)	2.32(1)	0.007(2)	12 (fix)	3.83(3)	0.012(4)	5.4(4)	140

(ii) Ceres 1 data were fitted: $2 \leq k \leq 9.0$ Å^{-1}, $1 \leq R \leq 5$ Å. These fits demonstrate the potential ligands at higher R. The structures used were: PuC, Pu_2C_3, PuO_2, $PuFe_2$ and δ-Pu – however it should be noted that most of these particular structures have XANES that are not in accordance with those measured for CeresI. CeresI XANES are typical of those of PuO.

**Pu** CeresI [408-409]	*n*C	*R*C (Å)	*n*O	*R*O (Å)	σ^2O= σ^2C (Å^2)	*n*Pu1	*R*Pu1 (Å)	*n*Pu3	*R*Pu3 (Å)	σ^2Pu(Å^2)	ΔE$_0$ (eV)	$\chi2_{red}$
Fit 1	6	2.29(3)	8	2.38(1)	0.002	8	3.17	12	3.48(3)	0.01	7.4(8)	209
Fit 2	2	2.50(11)	5.5	2.34(2)	0.002	8	3.17	12	3.49(3)	0.01	10(1)	202

(iii) Bruce Pu data were fitted: $2 \leq k \leq 8.0$ Å^{-1}, $1.3 \leq R \leq 4.2$ Å.

**Pu**	*n*O	*R*O (Å)	σ^2O (Å^2)†	*n*Ga	*R*Ga* (Å)	σ^2Ga (Å^2)	*n*Pu	*R*Pu (Å)	σ^2Pu(Å^2)	ΔE$_0$ (eV)	$\chi2_{red}$
Bruce-131506	4.9(7)	2.37(1)	0.017(4)	2.1(7)	3.36(3)	0.010 (fix)	2 (fix)	3.74(5)	0.010 (fix)	5.0(8)	9
Bruce-131398	6.4(9)	2.38(2)	0.024(4)							5.0(8)	9

*This peak can be fitted with a range of elements (Al, Ga, Pu) at a range of distances 2.96 to 3.36 Å. This may appear strange, but it is difficult to pin-point precisely which phase(s) is/are contributing to this peak.

†The EXAFS Debye-Waller term is very large for a first-shell Pu-O distance, suggesting disorder, ie this peak could be fitted with a range of Pu-O distances.

Values without errors were fixed during the fit.

Table S3. Qualitative μXRD TOPAS refinement of *Potatohead*.

**Scan**	***a*((U,Pu)O) Å**	***a*((Pu,U)O) Å**	**ΔV(%)**	**Refined wt% (UO_2)***	**Refined wt% (PuO_2)***	**R-factor**
131489	5.44745(23)	5.41948(25)	1.56	48(1)	52(1)	Rwp = 61
131490	5.44679(21)	5.41594 (20)	1.72	45(2)	59(1)	Rwp = 62
131491	5.44860(22)	5.41865(23)	1.67	40(1)	60(1)	Rwp = 60
131492	5.44895(23)	5.42019(25)	1.60	47(1)	52(1)	Rwp = 61
131493	5.44930(16)	5.41438(23(	1.95	55(1)	45(1)	Rwp = 58
131494	5.44514(21)	5.41506(21)	1.68	42(1)	58(1)	Rwp = 62
131496	5.45252(18)	5.42156(25)	1.72	57(1)	43(1)	Rwp = 58
AVERAGE	5.4484(23)	5.4179(28)	1.70			

*Normalized to 100%, but did not fit minor components - from FIB-SEM expect these to be Al-oxide or Al-Fe alloys.

Table S4. Particle U-L_3 edge EXAFS refinements.

U data were fitted with a combination of uraninite (predominantly U(IV)) and uranyl nitrate (U(VI)). Potatohead data were fitted: $2 \leq k \leq 10$ Å^{-1}, $1 \leq R \leq 5$ Å).

U	*n*O	*R*O (Å)	σ^2O (Å^2)	*n*U	*R*U (Å)	σ^2U(Å^2)	Phase %	ΔE_0 (eV)	$\chi 2_{red}$
Potatohead 479-480 **bulk**	8 (fix)<br>2 (fix)<br>2 (fix)<br>4 (fix)	2.31(1)<br>1.69(3)<br>2.30 (fix)<br>2.48(3)	0.014(1)<br>0.005 (fix)<br>0.014(1)<br>0.014(1)	12 (fix)	3.87(1)	0.009(1)	75 U(IV)<br>25 U(VI)	7.9(6)	125
Potatohead 483-484 **U-rich inclusion**	8 (fix)<br>2 (fix)<br>2 (fix)<br>2 (fix)<br>2xN (fix)†	2.37 (fix)<br>1.76(1)<br>2.40(2)<br>2.84(2)<br>2.24(2)	0.005(3)<br>0.005 (fix)<br>0.005(3)<br>0.005(3)<br>0.005(3)	12 (fix)	3.81(8)	0.021(16)	25 U(IV)<br>75 U(VI)	7.9(6)	125
Potatohead 486 **U-rich inclusion**	8 (fix)<br>2 (fix)<br>2 (fix)<br>2 (fix)<br>2xN (fix)†	2.37 (fix)<br>1.78(1)<br>2.18(3)<br>2.34(6)<br>2.42(8)	0.005 (fix)<br>0.005(3)<br>0.005 (fix)<br>0.005 (fix)<br>0.005 (fix)	12 (fix)	3.85(7)	0.005 (fix)	10(8) U(IV)<br>90(8) U(VI)	7.9(6)	125
Potatohead 498 **bulk**	8 (fix)<br>2 (fix)<br>2 (fix)<br>4 (fix)	2.35(1)<br>1.69(3)<br>2.17 (fix)<br>2.86 (fix)	0.011(3)<br>0.005 (fix)<br>0.011(3)<br>0.011(3)	12 (fix)	3.87(2)	0.007(3)	65(8) U(IV)<br>35(8) U(VI)	7.9(6)	125

*Phase% indicates the fitted amount of U(IV) (uraninite) and U(VI) (uranyl nitrate, $UO_2(NO_3)_2.6H_2O$).

† Fitted N – but this could be O in actual structure.

Table S5. Previous studies characterising Pu-bearing particles/colloids with imaging and/or XAS techniques. The studies are grouped by their broad geographical location.

**Study**	**Site**	**Source/Transport**	**No. of 'hot' particles**	**Technique**	**Summary of results**
Ikeda-Ohno et al. [5]	Maralinga, Australia	Nuclear & partial to non-fission weapons tests	1 particle from a large piece that broke into 5 particles.	SXRF<br>XANES+EXAFS	Pu and U distributions were de-coupled<br>$PuO_{2+x-y}(OH)_{2y}\cdot zH_2O$
Cooper et al. [3]<br>Burns et al. [2]	Maralinga, Australia	Nuclear & partial to non-fission weapons tests	5	PIXE	Pu and U distributed homogeneously in 2 samples (not detected in others) –areas scanned are Pu dominant
Batuk et al. [36]	McGuire Air Force Base, USA	Conventional/subcritical nuclear weapon accident	2	SXRF<br>XANES+EXAFS<br>XRD	homogenous and heterogenous Pu+U distribution<br>Pu-O, Pu-U-O and U-O phases<br>Fm3m diffraction pattern
Batuk et al. [36]	Los Alamos TA-21 waste site, USA	R&D waste	2	SXRF<br>XANES+EXAFS	Pu(IV) mononuclear species & Fe incorporated into Pu-O phase
Batuk et al. [36]	Rocky flats processing plant, Colorado, USA	Operational release	2	XANES+EXAFS<br>XRD	Pu-O, Pu-(U)-O phases
Batuk et al. [36]	Hanford, USA	Waste disposal site: Z-9 + Z12 cribs	3	SXRF<br>XANES+EXAFS<br>XANES+EXAFS	P incorporated into Pu-O phase<br>Pu(IV) mononuclear species

Kersting [44]	Hanford, USA	Waste disposal site: Z-9 crib	3?	Nano-SIMS	Pu colloids
Buck et al. [45]	Hanford, USA	Sediments Z9-4-5 and Z9-4-11 from Z (PFP) plant	2	SEM, TEM, EELS, EXAFS	Pu mainly present as PuO_2 + evidence for a nano-particulate Fe-Pu-phosphate phase. EXAFS shows P is O–bridged to the Pu in the PuO_2 lattice.
Felmy et al. [46]	Hanford, USA	Discharge from Z-9, Z-12 and subsurface sediments (Plutonium Finishing Plant- Z-plant complex)	4	EXAFS + XRD	Largest Pu particles found to be $PuO_{2+x-y}(OH)_{2y}.(H_2O)_{2z}$. Pu from sediment samples was either monomeric or highly disordered, with potential of F incorporated into PuO_2 structure.
Kersting et al. [47]	Nevada test site, USA	Atmospheric, ground & underground nuclear weapons tests	Solution	SEM XRD	Pu colloids in ground water
Varshney et al. [48]	Clean Slate II, Nevada test site, USA	Safety (nuclear weapon destruction) tests with chemical explosives	7	SEM-EDS	The authors note that particles were rougher with sharper edges (not "popcorn" or sponge-like as other non-critical accidents/releases); however, the images presented do not corroborate their statements.
Aragon et al. [49]	Palomares, Spain	Conventional/Non-critical nuclear weapon accident	7	SEM-EDX	Textures were typically massive and/or granulated with variable grain sizes to less than 0.5 µm; some particles fragmented. EDX showed primarily Pu and U with minor Si, Al, Fe and Cr.
Lind et al. [38]	Palomares, Spain	Conventional/Non-critical nuclear weapon accident	5	SEM-EDX+SXRF XANES	homogeneous Pu+U distribution Pu(III)+Pu(IV)+Pu(V) co-existing with U(IV)
Ranebo et al. [50]	Thule, Greenland	Conventional/Non-critical nuclear weapon accident	3	SEM-EDX, SIMS	Heterogeneous distribution of U and Pu in particles, with either a spongy or "popcorn" structure.
Lind et al. [39]	Thule, Greenland	Conventional/Non-critical nuclear weapon accident	3	SEM-EDX XANES	homogeneous Pu+U distribution

					Pu(III)+Pu(IV) homogeneously distributed with U(IV)
Erkisson et al. [37]	Thule, Greenland	Conventional/Non-critical nuclear weapon accident	5	SXRF imaging and u-SXRF-tomography<br>XANES	Pu occurs predominantly where the U occurs, however the Pu/U intensity distribution varies<br>2 particles contained predominantly Pu(IV) and 2 particles were 33% Pu(IV)+ 67 % Pu(VI); all were mixed heterogeneously with U(IV)
Jernstrom et al. [51]	Runit Island, Enewetak atoll, Marshall Islands	Nuclear test program included 4xair drops and 35x barge + 13xtower + 10xsurface + 3xunderwater detonations.	6	SXRF<br>SEM-EDX-WDX; SIMS	3xsmaller particles, containing mainly a Pu matrix, most likely from Quince safety test (based on $^{240}Pu/^{239}Pu$ & no ^{137}Cs).<br>3xlarger particles with a Si-O matrix containing heterogeneous distribution of Pu, most likely from the low-yield Fig test (based on $^{240}Pu/^{239}Pu$ & ^{137}Cs).
Erkisson et al. [37]	Muraroa atoll	Atmospheric & underground nuclear weapons tests	1	u-SXRF-tomography	Pu “nugget” attached to coral
Wolf et al. [52]	Johnston Atoll	Three non-nuclear destructs of nuclear warhead-carrying THOR<br>missiles in 1962	9	SEM-EDS+TEM-EDS<br>Electron diffraction	Pu and U are present as discrete phases in hot particles and EDS+electron diffraction provides evidence of $(U/Pu)O_2$, $(U/Pu)O_2CO_3$ and $(U/Pu)O_3•0.8H_2O$ phases in the soil.
Conway et al. [53]	Semipalatinsk NTS, former USSR	Low-yield (240 t), Pu-fuelled fission devices exploded at ~30 m below ground	9	SEM	‘Hot’ particles typically 1 mm in size with a vitrified, glass-like structure, typical of fused soil containing small discrete units of high-Z elements (Pu,Am – based on α-spectroscopy).
Novikov et al. [54]	Mayak, former USSR	Waste re-processing plant	Solution	nano-SIMS	Pu and U sorbed to iron-oxide colloids
Tamborini and Betti [55]	“Staryi Vishkov”, Novozybkov district, Russia	Certified soil IAEA-375 obtained from the International<br>Atomic Energy Agency	2	SEM, SIMS	SIMS/SEM was used to characterise Pu-containing particles with respect to size, shape and isotopic ratios and identified two different kinds of Pu; platelets of PuO_2 and fibrous PuO_2 rods.

Kurihara et al. (2020) [56]	Fukushima, Japan	Nuclear power plant accident due to 2011 earthquake and tsunami	3	SXRF, XANES, SEM, TEM	Pu(IV) detected in hot particles released from Fukushima accident.
Lewis et al. (2015)[41]	Unspecified	U-fuelled near-surface nuclear test	5	SEM, electron microprobe, nanoSIMS	Particles display chemical and textural heterogeneity
Wesiz et al. (2017)[43]	Unspecified	U-fuelled near-surface nuclear test	5	SEM, electron microprobe, nanoSIMS	Particles display chemical and textural heterogeneity
Holliday et al. (2017)[42]	Unspecified	Pu-fuelled near-surface nuclear test, no associated steel tower (not Trinity)	48	SEM	Significant chemical heterogeneity. Pu is preferentially associated with mafic glass compositions ($Si_{0.46}Ca_{0.28}Al_{0.16}Mg_{0.08}Fe_{0.02}O_{1.55}$.)
Pacold et al. (2016)[40]	Unspecified	• U-fuelled nuclear test containing some Pu (underground)<br>• near-surface nuclear test, primarily Pu-fuelled and containing some U<br>• near-surface, primarily U-fuelled nuclear test	3 (one from each test)	XANES+EXAFS	Mixed U(IV) & U(VI), Fe(II) present in all particles.<br>The particle from the primarily fuelled Pu test contained Pu(IV).

Supplementary Movies.

Movie S1: *Potatohead*. The reconstructed volume, in orange, corresponds to the pores and the blue corresponds to the envelope of the grain.

Movie S2: *Bruce* (a) slice from Fig. 1c and (b) slice from Fig. 1d. The reconstructed volume, in orange, corresponds to the Al-oxide-rich low-density phase and the blue corresponds to the envelope of the grain.

References.

1. Symonds, J. L., *A history of British atomic tests in Australia*. Australian Government Publishing Service: Australia, 1985.
2. Burns, P. A.; Cooper, M. B.; Williams, G. A.; Johnston, P. N. *Properties of Plutonium-Contaminated Particles Resulting from British Vixen B Trials at Maralinga*; Australian Radiation Laboratory: Victoria, 1990.
3. Cooper, M. B.; Burns, P. A.; Tracy, B. L.; Wilks, M. J.; Williams, G. A., Characterization of plutonium contamination at the former nuclear weapons testing range, at Maralinga in South Australia. *Journal of radioanalytical and nuclear chemistry* **1994,** *177*, (1), 161-184.
4. MARTAC, *Rehabilitation of former nuclear test sites at Emu and Maralinga (Australia).* Department of Education, Science and Training: 2003.
5. Ikeda-Ohno, A.; Shahin, L. M.; Howard, D. L.; Collins, R. N.; Payne, T. E.; Johansen, M. P., Fate of Plutonium at a Former Nuclear Testing Site in Australia. *Environ Sci Technol* **2016,** *50*, (17), 9098-104.
6. Kelly, S.; Hesterberg, D.; Ravel, B., Analysis of soils and minerals using X-ray absorption spectroscopy. In *Methods of Soil Analysis. Part 5. Mineralogical methods*, Soil Sciences Society of America: Madison, U.S.A, 2008; pp 398-463.
7. Solé, V. A.; Papillon, E.; Cotte, M.; Walter, P.; Susini, J., A multiplatform code for the analysis of energy-dispersive X-ray fluorescence spectra. *Spectrochimica Acta Part B: Atomic Spectroscopy* **2007,** *62*, (1), 63-68.
8. Coelho, A. A.; Evans, J.; Evans, I.; Kern, A.; Parsons, S., The TOPAS symbolic computation system. *Powder Diffraction* **2012,** *26*, (S1), S22-S25.
9. Ravel, B.; Newville, M., ATHENA, ARTEMIS, HEPHAESTUS: data analysis for X-ray absorption spectroscopy using IFEFFIT. *J Synchrotron Radiat* **2005,** *12*, (Pt 4), 537-41.
10. Rehr, J. J.; Kas, J. J.; Vila, F. D.; Prange, M. P.; Jorissen, K., Parameter-free calculations of X-ray spectra with FEFF9. *Phys Chem Chem Phys* **2010,** *12*, 5503-5513.
11. Conradson, S. D.; Abney, K. D.; Begg, B. D.; Brady, E. D.; Clark, D. L.; den Auwer, C.; Ding, M.; Dorhout, P. K.; Espinosa-Faller, F. J.; Gordon, P. L.; Haire, R. G., Higher order speciation effects on plutonium L_3 X-ray absorption near edge spectra. *Inorganic Chemistry* **2004,** *43*, (1), 116-131.
12. Conradson, S. D.; Begg, B. D.; Clark, D. L.; den Auwer, C.; Ding, M.; Dorhout, P. K.; Espinosa-Faller, F. J.; Gordon, P. L.; Haire, R. G.; Hess, N. J.; Hess, R. F.; Webster Keogh, D.; Morales, L. A.; Neu, M. P.; Paviet-Hartmann, P.; Runde, W.; Drew Tait, C.; Kirk Veirs, D.; Villella, P. M., Local and Nanoscale Structure and Speciation in the $PuO_{2+x-y}(OH)_{2y}\cdot zH_2O$ System. *Journal of the American Chemical Society* **2004,** *126*, (41), 13443-13458.

13. Syverson, D. D.; Etschmann, B.; Liu, W.; Ram, R.; Mei, Y.; Lanzirotti, T.; Mercadier, J.; Brugger, J., Oxidation state and coordination environment of Pb in U-bearing minerals. *Geochimica et Cosmochimica Acta* **2019,** *265*, 109-131.
14. Bunau, O.; Joly, Y., Time-dependent density functional theory applied to x-ray absorption spectroscopy. *Physical Review B* **2012,** *85*, (15).
15. Guda, S. A.; Guda, A. A.; Soldatov, M. A.; Lomachenko, K. A.; Bugaev, A. L.; Lamberti, C.; Gawelda, W.; Bressler, C.; Smolentsev, G.; Soldatov, A. V.; Joly, Y., Optimized Finite Difference Method for the Full-Potential XANES Simulations: Application to Molecular Adsorption Geometries in MOFs and Metal-Ligand Intersystem Crossing Transients. *J Chem Theory Comput* **2015,** *11*, (9), 4512-21.
16. Joly, Y.; Bunău, O.; Lorenzo, J. E.; Galéra, R. M.; Grenier, S.; Thompson, B., Self-consistency, spin-orbit and other advances in the FDMNES code to simulate XANES and RXD experiments. *Journal of Physics: Conference Series* **2009,** *190*.
17. Brugger, J.; Etschmann, B.; Liu, W.; Testemale, D.; Hazemann, J. L.; Emerich, H.; van Beek, W.; Proux, O., An XAS study of the structure and thermodynamics of Cu(I) chloride complexes in brines up to high temperature (400°C, 600bar). *Geochimica et Cosmochimica Acta* **2007,** *71*, (20), 4920-4941.
18. Etschmann, B. E.; Mei, Y.; Liu, W.; Sherman, D.; Testemale, D.; Müller, H.; Rae, N.; Kappen, P.; Brugger, J., The role of Pb(II) complexes in hydrothermal mass transfer: An X-ray absorption spectroscopic study. *Chemical Geology* **2018,** *502*, 88-106.
19. Atwood, R. C.; Bodey, A. J.; Price, S. W.; Basham, M.; Drakopoulos, M., A high-throughput system for high-quality tomographic reconstruction of large datasets at Diamond Light Source. *Philos Trans A Math Phys Eng Sci* **2015,** *373*, (2043).
20. Schindelin, J.; Arganda-Carreras, I.; Frise, E.; Kaynig, V.; Longair, M.; Pietzsch, T.; Preibisch, S.; Rueden, C.; Saalfeld, S.; Schmid, B.; Tinevez, J. Y.; White, D. J.; Hartenstein, V.; Eliceiri, K.; Tomancak, P.; Cardona, A., Fiji: an open-source platform for biological-image analysis. *Nat Methods* **2012,** *9*, (7), 676-82.
21. Group, F. AVIZO 3D Analysis Software.
22. Hund, V. F., Fluorite Mixed Phases of Uranium, Thorium, Cerium, and Zirconium Dioxide with Bismuth Oxide. *Zeitschrift für anorganische und allgemeine Chemie* **1964,** *333*, 248-255.
23. Zachariasen, W. H., Crystal chemical studies of the 5f-series of elements. XII. New compounds representing known structure types. *Acta Crystallography* **1949,** *2*, 388-390.
24. Ellinger, F. H.; Zachariasen, W. H., The crystal structures of PuGa4 and PuGa6. *Acta Crystallography* **1965,** *19*, 281-283.
25. Ellinger, F. H.; Land, C. C.; Struebing, V. O., The Plutonium-Gallium system. *Journal of Nuclear Materials* **1964,** *12*, (2), 226-236.
26. Tucker, P. A.; Etter, D. E.; Gebhart, J. M., Phase equilibria in the ternary system Pu-Ce-Fe. In *Plutonium 1965, Proc. Int. Conf*, Kay, A. E.; Waldron, M. B., Eds. Chapman and Hall, London: 1965; pp 392-404.
27. Larson, A. C.; Cromer, D. T.; Stambaugh, C. K., The crystal structure of PuAl3. *Acta Crystallography* **1957,** *10*, 443-446.
28. Runnalls, O. J. C., Phase Equilibria Studies on the Aluminum-Plutonium System. In *Plutonium 1965, Proc. Int. Conf*, Kay, A. E.; Waldron, M. B., Eds. Chapman and Hall, London: 1965; pp 341-357.

29. Bean, A. C.; Abney, K.; Scott, B. L.; Runde, W., Structural Characterization of the First Hydroxo-Bridged Plutonium Compound, $(PuO_2)_2(IO_3)(\mu_2\text{-}OH)_3$. *Inorganic Chemistry* **2005,** *44*, 5209-5211.
30. Cotton, S., *Lanthanide and Actinide Chemistry*. John Wiley & Sons: Rutland, UK, 2006.
31. Sina, H.; Corneliusson, J.; Turba, K.; Iyengar, S., A study on the formation of iron aluminide (FeAl) from elemental powders. *Journal of Alloys and Compounds* **2015,** *636*, 261-269.
32. Nelson, A. J.; Felter, T. E.; Wu, K. J.; Evans, C.; Ferreira, J. L.; Siekhaus, W. J.; McLean, W., Uranium passivation by C+ implantation: A photoemission and secondary ion mass spectrometry study. *Surface Science* **2006,** *600*, (6), 1319-1325.
33. Lind, O. C.; Salbu, B.; Skipperud, L.; Janssens, K.; Jaroszewicz, J.; De Nolf, W., Solid state speciation and potential bioavailability of depleted uranium particles from Kosovo and Kuwait. *Journal of environmental radioactivity* **2009,** *100*, (4), 301-307.
34. Clark, D. L.; Hecker, S. S.; Jarvinen, G. D.; Neu, M. P., Plutonium. In *The chemistry of the Actinide and Transactinide Elements*, 4th ed.; Morss, L. R.; Edelstein, N. M.; Fuger, J., Eds. Springer: 2010; pp 813-1264.
35. Fox, A. R.; Bart, S. C.; Meyer, K.; Cummins, C. C., Towards uranium catalysts. *Nature* **2008,** *455*, (7211), 341-9.
36. Batuk, O. N.; Conradson, S. D.; Aleksandrova, O. N.; Boukhalfa, H.; Burakov, B. E.; Clark, D. L.; Czerwinski, K. R.; Felmy, A. R.; Lezama-Pacheco, J. S.; Kalmykov, S. N.; Moore, D. A.; Myasoedov, B. F.; Reed, D. T.; Reilly, D. D.; Roback, R. C.; Vlasova, I. E.; Webb, S. M.; Wilkerson, M. P., Multiscale Speciation of U and Pu at Chernobyl, Hanford, Los Alamos, McGuire AFB, Mayak, and Rocky Flats. *Environmental Science & Technology* **2015,** *49*, (11), 6474-6484.
37. Eriksson, M.; Osán, J.; Jernström, J.; Wegrzynek, D.; Simon, R.; Chinea-Cano, E.; Markowicz, A.; Bamford, S.; Tamborini, G.; Török, S.; Falkenberg, G.; Alsecz, A.; Dahlgaard, H.; Wobrauschek, P.; Streli, C.; Zoeger, N.; Betti, M., Source term identification of environmental radioactive Pu/U particles by their characterization with non-destructive spectrochemical analytical techniques. *Spectrochimica Acta Part B: Atomic Spectroscopy* **2005,** *60*, (4), 455-469.
38. Lind, O. C.; Salbu, B.; Janssens, K.; Proost, K.; Garcia-Leon, M.; Garcia-Tenorio, R., Characterization of U/Pu particles originating from the nuclear weapon accidents at Palomares, Spain, 1966 and Thule, Greenland, 1968. *Sci Total Environ* **2007,** *376*, (1-3), 294-305.
39. Lind, O. C.; Salbu, B.; Janssens, K.; Proost, K.; Dahlgaard, H., Characterization of uranium and plutonium containing particles originating from the nuclear weapons accident in Thule, Greenland, 1968. *J Environ Radioact* **2005,** *81*, (1), 21-32.
40. Pacold, J. I.; Lukens, W. W.; Booth, C. H.; Shuh, D. K.; Knight, K. B.; Eppich, G. R.; Holliday, K. S., Chemical speciation of U, Fe, and Pu in melt glass from nuclear weapons testing. *Journal of Applied Physics* **2016,** *119*, (19).
41. Lewis, L. A.; Knight, K. B.; Matzel, J. E.; Prussin, S. G.; Zimmer, M. M.; Kinman, W. S.; Ryerson, F. J.; Hutcheon, I. D., Spatially-resolved analyses of aerodynamic fallout from a uranium-fueled nuclear test. *J Environ Radioact* **2015,** *148*, 183-95.
42. Holliday, K. S.; Dierken, J. M.; Monroe, M. L.; Fitzgerald, M. A.; Marks, N. E.; Gostic, R. C.; Knight, K. B.; Czerwinski, K. R.; Hutcheon, I. D.; McClory, J. W., Plutonium segregation in glassy aerodynamic fallout from a nuclear weapon test. *Dalton Trans* **2017,** *46*, (6), 1770-1778.

43. Weisz, D. G.; Jacobsen, B.; Marks, N. E.; Knight, K. B.; Isselhardt, B. H.; Matzel, J. E.; Weber, P. K.; Prussin, S. G.; Hutcheon, I. D., Deposition of vaporized species onto glassy fallout from a near-surface nuclear test. *Geochimica et Cosmochimica Acta* **2017,** *201*, 410-426.
44. Kersting, A. B., Plutonium transport in the environment. *Inorg Chem* **2013,** *52*, (7), 3533-46.
45. Buck, E. C.; Moore, D. A.; Czerwinski, K. R.; Conradson, S. D.; Batuk, O. N.; Felmy, A. R., Nature of nano-sized plutonium particles in soils at the Hanford Site. *Radiochimica Acta* **2014,** *102*, (12).
46. Felmy, A. R.; Cantrell, K. J.; Conradson, S. D., Plutonium contamination issues in Hanford soils and sediments: Discharges from the Z-Plant (PFP) complex. *Physics and Chemistry of the Earth, Parts A/B/C* **2010,** *35*, (6-8), 292-297.
47. Kersting, A. B.; Efurd, D. W.; Finnegan, D. L.; Rokop, D. J.; Smith, D. K.; Thompson, J. L., Migration of plutonium in ground water at the Nevada test site. *Nature* **1999,** *397*, (6714), 56-59.
48. Varshney, G.; Cezeaux, J. R.; Petrosky, J. C., Investigation of fissile materials collected from a non-critical nuclear explosion site using non-destructive analytical techniques. *Journal of Radioanalytical and Nuclear Chemistry* **2018,** *318*, (1), 505-513.
49. Aragon, A.; Espinosa, A.; de la Cruz, B.; Fernandez, J. A., Characterization of radioactive particles from the Palomares accident. *J Environ Radioact* **2008,** *99*, (7), 1061-7.
50. Ranebo, Y.; Eriksson, M.; Tamborini, G.; Niagolova, N.; Bildstein, O.; Betti, M., The use of SIMS and SEM for the characterization of individual particles with a matrix originating from a nuclear weapon. *Microsc Microanal* **2007,** *13*, (3), 179-90.
51. Jernström, J.; Eriksson, M.; Simon, R.; Tamborini, G.; Bildstein, O.; Marquez, R. C.; Kehl, S. R.; Hamilton, T. F.; Ranebo, Y.; Betti, M., Characterization and source term assessments of radioactive particles from Marshall Islands using non-destructive analytical techniques. *Spectrochimica Acta Part B: Atomic Spectroscopy* **2006,** *61*, (8), 971-979.
52. Wolf, S. F.; Bates, J. K.; Buck, E. C.; Dietz, N. L.; Fortner, J. A.; Brown, N. R., Physical and chemical characterization of actinides in soil from Johnston Atoll. *Environmental Science & Technology* **1997,** *31*, (2), 467-471.
53. Conway, M.; Leon Vintro, L.; Mitchell, P. I.; Garcia-Tenorio, R.; Jimenez-Ramos, M. C.; Burkitbayev, M.; Priest, N. D., In-vitro analysis of the dissolution kinetics and systemic availability of plutonium ingested in the form of 'hot' particles from the Semipalatinsk NTS. *Appl Radiat Isot* **2009,** *67*, (5), 884-8.
54. Novikov, A. P.; Kalmykov, S. N.; Utsunomiya, S.; Ewing, R. C.; Horreard, F.; Merkulov, A.; Clark, S. B.; Tkachev, V. V.; Myasoedov, B. F., Colloid Transport of Plutonium in the Far-Field of the Mayak Production Association, Russia. *Science* **2006,** *314*, (5799), 638-641.
55. Tamborini, G.; Betti, M., Characterisation of radioactive particles by SIMS. *Microchimica Acta* **2000,** *132*, (2-4), 411-417.
56. Kurihara, E.; Takehara, M.; Suetake, M.; Ikehara, R.; Komiya, T.; Morooka, K.; Takami, R.; Yamasaki, S.; Ohnuki, T.; Horie, K.; Takehara, M.; Law, G. T. W.; Bower, W.; JF, W. M.; Warnicke, P.; Grambow, B.; Ewing, R. C.; Utsunomiya, S., Particulate plutonium released from the Fukushima Daiichi meltdowns. *Sci Total Environ* **2020,** *743*, 140539.
